# *Lawsonia intracellularis* infected enterocytes lack sucrase-isomaltase which contributes to reduced pig digestive capacity

**DOI:** 10.1186/s13567-021-00958-2

**Published:** 2021-06-19

**Authors:** Emma T. Helm, Eric R. Burrough, Fernando L. Leite, Nicholas K. Gabler

**Affiliations:** 1 Department of Animal Science, Iowa State University, Ames, IA 50011 USA; 2 Department of Veterinary Diagnostic and Production Animal Medicine, Iowa State University, Ames, IA 50011 USA; 3 Boehringer Ingelheim Animal Health USA Inc, Duluth, GA 30096 USA

**Keywords:** *Lawsonia intracellularis*, Vaccine, Cell proliferation, Notch signaling, Wnt signaling, Digestibility, Intestinal integrity

## Abstract

*Lawsonia intracellularis* is endemic to swine herds worldwide, however much is still unknown regarding its impact on intestinal function. Thus, this study aimed to characterize the impact of *L. intracellularis* on digestive function, and how vaccination mitigates these impacts. Thirty-six *L. intracellularis* negative barrows were assigned to treatment groups (*n * =  12/trt): (1) nonvaccinated, *L. intracellularis* negative (NC); (2) nonvaccinated, *L intracellularis* challenged (PC); and (3) *L. intracellularis* challenged, vaccinated (Enterisol^®^ Ileitis, Boehringer Ingelheim) 7 weeks pre-challenge (VAC). On days post-inoculation (dpi) 0 PC and VAC pigs were inoculated with *L. intracellularis*. From dpi 19–21 fecal samples were collected for apparent total tract digestibility (ATTD) and at dpi 21, pigs were euthanized for sample collection. Post-inoculation, ADG was reduced in PC pigs compared with NC (41%, *P * <  0.001) and VAC (25%, *P * <  0.001) pigs. Ileal gross lesion severity was greater in PC pigs compared with NC (*P * =  0.003) and VAC (*P * =  0.018) pigs. Dry matter, organic matter, nitrogen, and energy ATTD were reduced in PC pigs compared with NC pigs (*P * ≤  0.001 for all). RNAscope in situ hybridization revealed abolition of sucrase-isomaltase transcript in the ileum of PC pigs compared with NC and VAC pigs (*P * <  0.01). Conversely, abundance of stem cell signaling markers Wnt3, Hes1, and p27^Kip1^ were increased in PC pigs compared with NC pigs (*P * ≤  0.085). Taken together, these data demonstrate that reduced digestibility during *L. intracellularis* challenge is partially driven by abolition of digestive machinery in lesioned tissue. Further, vaccination mitigated several of these effects, likely from lower bacterial burden and reduced disease severity.

**Supplementary Information:**

The online version contains supplementary material available at 10.1186/s13567-021-00958-2.

## Introduction

Understanding and managing sub-clinical and clinical pathogenic disease in swine remains a formidable challenge for pork producers worldwide. One enteric pathogen of particular concern to pork producers is *Lawsonia intracellularis,* an intracellular bacterium responsible for proliferative enteropathy in growing pigs [1]. The bacterium is endemic to swine farms, with worldwide farm presence approaching 96%, and is responsible for reductions in growth upwards of 60% in clinically affected pigs [2–4].

Due to its obligate intracellular nature, understanding the interaction of *L. intracellularis* with the host is critical to fully understanding pathogenesis of this disease. The bacterium primarily infects enterocytes of the terminal ileum, although infected enterocytes may extend proximally into the jejunum or distally into the large intestine [5, 6]. Upon establishing within infected epithelial enterocytes, *L. intracellularis* induces proliferation of undifferentiated epithelial cells, resulting in mucosal hyperplasia [1]. Additionally, *L. intracellularis* infection is associated with impaired nutrient absorption in hamsters, suggesting there may be dysregulation in the pathways that lead to functional absorptive cells [7]. In pigs, peak bacterial infection is associated with reduced abundance of genes related to mucosal integrity and cellular transport [8–10], suggesting these factors may be a causation for reduced growth and feed efficiency during *L. intracellularis* infection. In particular, Vannucci et al. [8] observed consistent downregulation of genes associated with nutrient acquisition and sucrose degradation, including the brush border disaccharidase sucrose-isomaltase. However, the functional implications of these gene abundance changes, specifically regarding intestinal integrity and digestive and absorptive function, remains unclear.

Historically, antimicrobials were widely used to control *L. intracellularis* [1]. With more judicious use of antibiotics by the swine industry, vaccination has emerged as a key strategy to mitigate disease [1]. Vaccination of pigs with a live, attenuated vaccine has been shown to reduce bacterial fecal shedding and improve growth performance compared with non-vaccinated pigs [11]. A partial reason for the improvement in growth may be greater intestinal barrier integrity and digestive and absorptive function. However, there is little controlled study data available regarding this physiology. Thus, this study aimed to understand the impact of a *L. intracellularis* challenge on growth performance, disease severity, and intestinal integrity and digestive function in non-vaccinated and pigs vaccinated with a live, attenuated *L. intracellularis* vaccine.

## Materials and methods

All animal procedures were approved by the Iowa State University Institutional Animal Care and Use Committee (IACUC protocol #19-170) and adhered to the ethical and humane use of animals for research.

### Animals, housing, and experimental design

A total of 51 newly weaned barrows were randomly selected from a high health herd with no known history of *L. intracellularis* that had been vaccinated for porcine circovirus type 2 and *Mycoplasma hyopneumoniae* (Midwest Research Swine, Gibbon, MN, USA). At 21 days of age (weaning), pigs were confirmed negative for *L. intracellularis* pathogen and antibodies via individual fecal PCR and serum antibody ELISA and were transported to an isolated facility for housing during the nursery phase. At 1-week post-weaning, a cohort of 20 barrows of average weight were randomly selected to be vaccinated with Enterisol^®^ Ileitis (Boehringer Ingelheim Animal Health, Duluth, GA, USA) via 1 mL oral drench. These pigs were housed in a separate barn for the remainder of the nursery phase to prevent exposure of non-vaccinated pigs to the modified-live vaccine strain.

At 7 weeks post-weaning, 12 vaccinated and 24 nonvaccinated pigs (34  ±  2.3 kg BW) were selected from these 2 cohorts, excluding pigs with highest and lowest body weights, and transported to Ames, IA. These 36 pigs were assigned to individual pens across two rooms in the same barn and assigned to individual treatment groups as follows (*n * =  12 pigs/trt): (1) nonvaccinated, *L. intracellularis* negative (NC); (2) nonvaccinated, *L intracellularis* challenged (PC); and (3) vaccinated, *L. intracellularis* challenged (VAC). The NC pigs were housed in a separate room to prevent potential pathogen spread. The two rooms had identical pen size, feeders, flooring, heating, cooling, and water supply, but separate manure pits. All pigs were ad libitum fed the same diet throughout the experiment, which contained no antimicrobials and met or exceeded all NRC [12] requirements (Additional file 1). After a 1-week acclimation, on days post-inoculation (dpi) 0 and 7 weeks after vaccination of VAC pigs, PC and VAC pigs were inoculated with *L. intracellularis* via gastric gavage (2.7 × 10^8^ organisms/mL; determined by quantitative PCR). The *L. intracellularis* inoculum was an intestinal homogenate collected from a lesioned *L. intracellularis* positive pig obtained through a commercial supplier (Gutbugs Inc, Fergus Falls, MN, USA). The inoculum was tested previously by the supplier to be negative for presence of different pathogens such as *Salmonella enterica,* porcine respiratory and reproductive syndrome virus, enterotoxigenic *Escherichia coli*, coccidian oocysts, and nematode eggs. Individual feed disappearance and BW were recorded for each pig on dpi 0, 7, 14, and 19. From these recordings, individual pig average daily gain (ADG), average daily feed intake (ADFI), and feed efficiency (Gain:Feed; G:F) were calculated.

Fecal swabs were collected on all pigs at dpi 0, 7, 14, and at necropsy. Swabs were submitted to the Iowa State Veterinary Diagnostic Lab (ISU VDL) for quantitative PCR to evaluate *L. intracellularis* fecal shedding. On dpi 0, 7, 14, and necropsy, blood samples (10 mL) were collected on all pigs. Blood samples were collected into BD Serum Vacutainer tubes (Becton, Dickinson and Company, Franklin Lakes, NJ, USA) via jugular venipuncture. Samples were centrifuged (2000 × *g* for 10 min at 4 °C), and serum was collected, aliquoted, and stored at  −80 °C until analysis. One aliquot was submitted to the ISU VDL prior to freezing to quantify *L. intracellularis* antibody response via the SVANOIR Ileitis ELISA (Boehringer Ingelheim Svanova, Uppsala, Sweden).

Pigs were euthanized at approximately dpi 21 (dpi 19–23) for luminal content and tissue collection. Pigs were euthanized in reps of 6–8 pigs per rep, with at least 2 NC pigs included in each necropsy rep. Necropsies were performed over several days to allow for completion of the fresh tissue assays outlined herein. Repetition of necropsy was initially included in the statistical model, however was removed as it did not have an appreciable effect on variables of interest. Pigs were euthanized by captive bolt followed by exsanguination. Sections from the terminal ileum and apex of the spiral colon were rinsed in Krebs buffer (25 mM NaHCO_3_, 120 mM NaCl, 1 mM MgSO_4_, 6.3 mM KCl, 2 mM CaCl_2_, and 0.32 mM NaH_2_PO_4_) and placed in continuously aerated bottles containing Krebs buffer for transport to the laboratory for analysis. Additional sections of the distal ileum, cecum, and spiral colon were placed in neutral buffered formalin, and samples of ileal mucosal scrapings were preserved in RNAlater (Thermofisher Scientific, Waltham, MA, USA) for 24 h prior to storage at  −80 °C.

### Gross pathology

At necropsy, the entire jejunum, ileum, cecum, and colon were examined and scored for gross lesions characteristic of enteric disease and *L. intracellularis* infection. Macroscopic lesions were evaluated on a scale of 0–4 as follows: 0  =  no gross lesions; 1  =  mild edema and hyperemia of mucosa or serosa; 2  =  edema, hyperemia, multifocal reticulated mucosa (thickening); 3  =  edema, hyperemia, reticulated appearance of the serosa and thickening of the mucosa; and 4 if severe mucosal thickening, luminal hemorrhage, or mucosal necrosis. Additionally, the length of the lesioned area in the ileum was measured and recorded.

### Microscopic pathology and morphology

The distal ileum, cecal apex, and apex of spiral colon were fixed in 10% neutral buffered formalin, then trimmed, processed, and sectioned at the ISU VDL for histopathologic analysis by a blinded, board-certified veterinary pathologist at the ISU VDL. Sections were either hematoxylin and eosin stained or immunohistochemically stained (IHC) for *L. intracellularis* using routine methods at the ISU VDL. *L. intracellularis* IHC was evaluated on a scale of 0–4 scale as follows: 0  =  no *L. intracellularis* antigen, 1  =  0–25% of enterocytes had detectible antigen; 2  =  25–50% of enterocytes were positive with antigen; 3  =  50–75% of enterocytes were positive; and 4  =  75–100% of enterocytes were positive with antigen [7].

Hematoxylin and eosin slides were evaluated for several parameters characteristic of *L. intracellularis* infection. Slides were evaluated for the overall presence of microscopic lesions on a 4-point scale as follows: 0  =  no lesion; 1  =  focal lesions; 2  =  multifocal lesions; and 3  =  diffuse lesions. Inflammation (evidence of infiltrating inflammatory immune cells) was scored on a 4-point scale as follows: 0  =  no/minimal inflammation; 1  =  mild inflammation; 2  =  moderate inflammation; and 3  =  severe inflammation. Increases in crypt epithelial hyperplasia were evaluated on 4-point scale as follows: 0  =  none/minimal; 1  =  mild; 2  =  moderate; and 3  =  severe. Hematoxylin and eosin slides were also used to evaluate intestinal morphology. Images were taken at 4 ×  magnification using a DP80 Olympus Camera mounted on an OLYMPUS BX 53/43 microscope (Olympus Scientific, Waltham, MA, USA), and 15 well orientated villus and crypt pairs (ileum) or 15 crypts (cecum and colon) were measured using OLYMPUS CellSens Dimension 1.16 software (Olympus Scientific) as previously described [13].

### RNA Chromogenic in-situ hybridization

Visualization of mRNA transcripts was performed using RNAScope^®^ 2.5 (Advanced Cell Diagnostics, Hayward, CA, USA), according to the manufacturer’s instructions at the ISU VDL. *Sus scrofa*-specific proprietary probe combinations were used for sucrase-isomaltase (brown) and Hes1, a component of the Notch signaling pathway indicative of cells predestined to become absorptive enterocytes [14] (red; Advanced Cell Diagnostics, Hayward, CA, USA). Slides were imaged at 40 ×  magnification with a DP80 Olympus Camera mounted on an OLYMPUS BX 53/43 microscope (Olympus Scientific). Three images were taken per slide to acquire approximately 8–9 well orientated villi per pig. Individual villi and their adjacent crypts were split equally along the villus-crypt axis into three separate regions of interest: crypts, mid-villi, and villus tips, and the epithelial layer was outlined within each region. Images were analyzed using the RNA in-situ hybridization module of HALO image analysis software (HALO™, Indica Labs, Inc., Corrales, NM, USA). The module identified chromogenic duplex signals (red or brown) and these signals were quantified. Due to the overwhelming intensity of the brown sucrase-isomaltase signal in control tissues, Hes1 stain was unable to be accurately semi-quantified. Sucrase-isomaltase signal was quantified as the percent positive stain area within the region of interest, normalized to total area of each region of interest.

### Ileal cytokine analysis

Ileal cytokine concentrations were determined in protein extracted from frozen ileum tissues. Briefly, tissues (0.5 g) were homogenized in Tris–HCl lysis buffer [0.05% Tween-20, 0.1% protease inhibitor cocktail, 20 mM Tris–HCl (pH 7.5), and 150 mM NaCl], centrifuged (2000 × *g* for 10 min at 4 °C), and protein concentrations of the supernatant were determined with a bicinchoninic acid (BCA) assay (Thermofisher Scientific). Protein extracts were adjusted to 2 mg/mL, from which 50 µg was loaded into each well for the cytokine assay. Cytokine concentrations were determined using a commercially available, bead-based immunoassay validated for use in pigs (MILLIPLEX_MAP_ Porcine Cytokine/Chemokine panel kit, Millipore Sigma, Burlington, MA, USA). Samples were read with a Luminex MAGPIX^®^ Multiplex Reader (Millipore Sigma), and data are presented as pg/mg isolated ileum protein.

### Ileal mRNA extraction and RT-PCR

Total mRNA was extracted from RNAlater preserved ileum scrapings with a Direct-zol RNA Miniprep Kit (Zymo Research, Irvine, CA, USA). Quantity and purity of extracted mRNA was determined spectrophotometrically using a Cytation 5 Hybrid Multi-Mode Reader (BioTek Instruments Inc., Winooski, VT, USA). All samples had a 260/280 ratio of at least 1.8. One thousand nanograms of mRNA was transcribed with a commercially available kit (Thermofisher Scientific) and cDNA was used for real-time PCR using iQ SYBR Green Supermix (Bio-Rad Laboratories, Inc., Hercules, CA) and an iQ5 Optical System (Bio-Rad Laboratories). Abundance values were normalized to a reference gene (*ACTB*) and NC pigs according to the 2^−ΔΔCt^ method. Gene symbols and primer sequences are listed in Additional file 2.

### Ex vivo assessment of barrier function and integrity

Fresh ileum and colon sections transported in Krebs buffer were mounted in modified Ussing chambers within approximately 1–1.5 h of euthanasia. Modified Ussing chambers were assembled and electrophysiological and fluorescein isothiocyanate-dextran 4 kDa (FD4) macromolecule permeability measurements were collected as described previously [15]. Estimates of active glucose and glutamine transport for ileal samples were calculated as the change in current (µA) after nutrient addition. A fluorescent plate reader (Cytation 5 Hybrid Multi-Mode Reader, BioTek Instruments Inc.,) was used to determine changes in relative fluorescence of FD4 in the serosal samples from 0 to 60 min after FD4 addition at 485 and 520 nm excitation and emission wavelengths, respectively.

To further assess barrier permeability, mucosal to serosal translocation of *Salmonella enterica* serovar Typhimurium (*S.* Typhimurium) was determined in the ileum and colon. Each mucosal chamber was spiked with 400 μL nalidixic acid resistant *S.* Typhimurium strain 798 provided by Dr. Richard Isaacson (Optical density at 600 nm = 2.0, approximately 6 × 10^12^ colony forming units/mL). One hour after adding *S.* Typhimurium to the mucosal chamber, 1 mL sample was removed from the serosal chamber, serially diluted, and plated onto brilliant green agar plates containing nalidixic acid. Plates were incubated at 37 ℃ for 24 h. Nalidixic acid resistant *S.* Typhimurium colonies were counted, multiplied by the dilution factor, and expressed as colony forming units per mL (CFUs/mL).

### Mitochondrial isolation, reactive oxygen species production, and oxygen consumption

Mitochondria were isolated from ileum and colon tissue via differential centrifugation as previously described [16]. Washed mitochondria were resuspended in 3 mL mitochondrial wash buffer, protein concentrations were determined via BCA assay (Thermofisher Scientific), and were diluted to a protein concentration of 2 mg/mL and stored at 4 ℃ until use [16].

Mitochondrial reactive oxygen species (ROS) production was determined in isolated mitochondria using a 2′,7′-Dichlorofluorescin diacetate (DCFH) assay described previously [16–18]. Mitochondrial hydrogen peroxide production was calculated from a hydrogen peroxide standard curve based on fluorescence values of DCFH. Plates were incubated at 37 °C and read at 0, 5, 10, 15, and 20 min after adding the energy substrate. Readings were used to calculate the rate of hydrogen peroxide production per min, expressed as μmol hydrogen peroxide produced/mg mitochondrial protein/min.

Mitochondrial oxygen consumption was evaluated using a Seahorse XFe24 Extracellular Flux Analyzer (Seahorse Bioscience, North Billerica, MA, USA) as previously described [19–21]. Mitochondria (40 μg protein) were plated into a V7 XFe24 Tissue Culture Plate, diluted with mitochondrial assay buffer (220 mM Mannitol, 70 mM Sucrose, 5 mM KH_2_PO_4_, 5 mM MgCl_2_, 2 mM HEPES, 1 mM EGTA, 0.5 mg/mL BSA, pH 7.4), and centrifuged (2000 × *g* for 10 min at 4 °C). Thereafter, 450 μL substrate buffer (mitochondrial assay buffer  +  5.5 mM glutamate  +  5.5 mM malate, pH 7.4) was added to each well. The plate was incubated at 37 ℃ for 8–10 min and then transferred to the XFe24 instrument for the experiment, as previously described [16]. Data were then presented as pmoles O_2_ per min.

### Apparent ileal and total tract digestibility

A representative feed sample from the complete diet was obtained for analysis. Fecal samples were collected from all pigs over 3 consecutive days (17–19 dpi). Additionally, digesta from the distal ileum and cecum were collected at necropsy. Fecal and digesta samples were stored at  −20 ℃ until further analysis. Fecal samples were thawed, homogenized within pig, and dried in a mechanical confection oven at 100 ℃. Digesta samples were freeze dried (Labcono Bulk Tray Dryer, Labcono Corp., Kansas City, MO, USA). Feed samples were ground through a 2-mm screen (Model ZM1; Retsch Inc., Newton, PA, USA) while digesta and fecal samples were ground with a mortar and pestle. Proximate analysis was performed on feed, feces, and digesta as previously described by Schweer et al. [22]. All samples were analyzed for dry matter (DM; AOAC method 930.15), titanium dioxide [23], nitrogen (N; TruMac N, Leco Corporation, St. Joseph, MO, USA), and gross energy (GE) using bomb calorimetry (Oxygen Bomb Calorimetry 6200; Parr Instruments, Moline, IL, USA). Organic matter (OM) was determined with the ashing method and calculated as described previously [24]. Apparent ileal digestibility (AID), apparent cecal digestibility (ACD), and apparent total tract digestibility (ATTD) coefficients for DM, OM, N, and GE were calculated using the index method [25].

### Statistical analysis

Statistical analysis of all data was performed in SAS 9.4 (SAS Institute, Cary, NC, USA). The following mixed model was fitted to quantitative parameters:

$${\text{Y}}_{{ij}} {\text{ = }}\mu {\text{ + TRT}}_{i} {\text{ + e}}_{{ij}}$$

wherein Y_ij_  =  the phenotype measured on animal *j*; Trt_i_  =  effect of treatment (fixed effect; NC, PC, VAC); and e_ij_  =  error term of animal *k* subjected to treatment *i*, e_ij_  ~  N (0, σ_e_^2^). Least square means were determined using the LS means statement and differences in LS means were produced with the pdiff option. *S.* Typhimurium translocation data were log transformed and analyzed in the GLIMMIX procedure, assuming a negative binomial distribution. Serum antibody levels and fecal bacterial counts were analyzed using the above model with the inclusion of a repeated measures statement, with variance–covariance models determined based on evaluation of fit statistics [the (corrected) Akaike’s information criterion and the Sawa Bayesian information criterion] for each individual trait. These data are presented as Least Squares means with a pooled standard error. Binary categorical lesion scores were analyzed using Chi-square tests and *P *values for pairwise comparisons were corrected using Bonferroni adjustments. Multinomial categorial lesion scores were analyzed with Kruskal–Wallis tests in the NPAR1WAY procedure, with *P *values adjusted for pairwise comparisons. For all analyses, differences were considered significant when *P * ≤  0.05 and a tendency when 0.05  <  *P * ≤  0.10.

## Results

### Clinical observations, antibody levels and fecal shedding, lesion scoring

**Figure 1:**
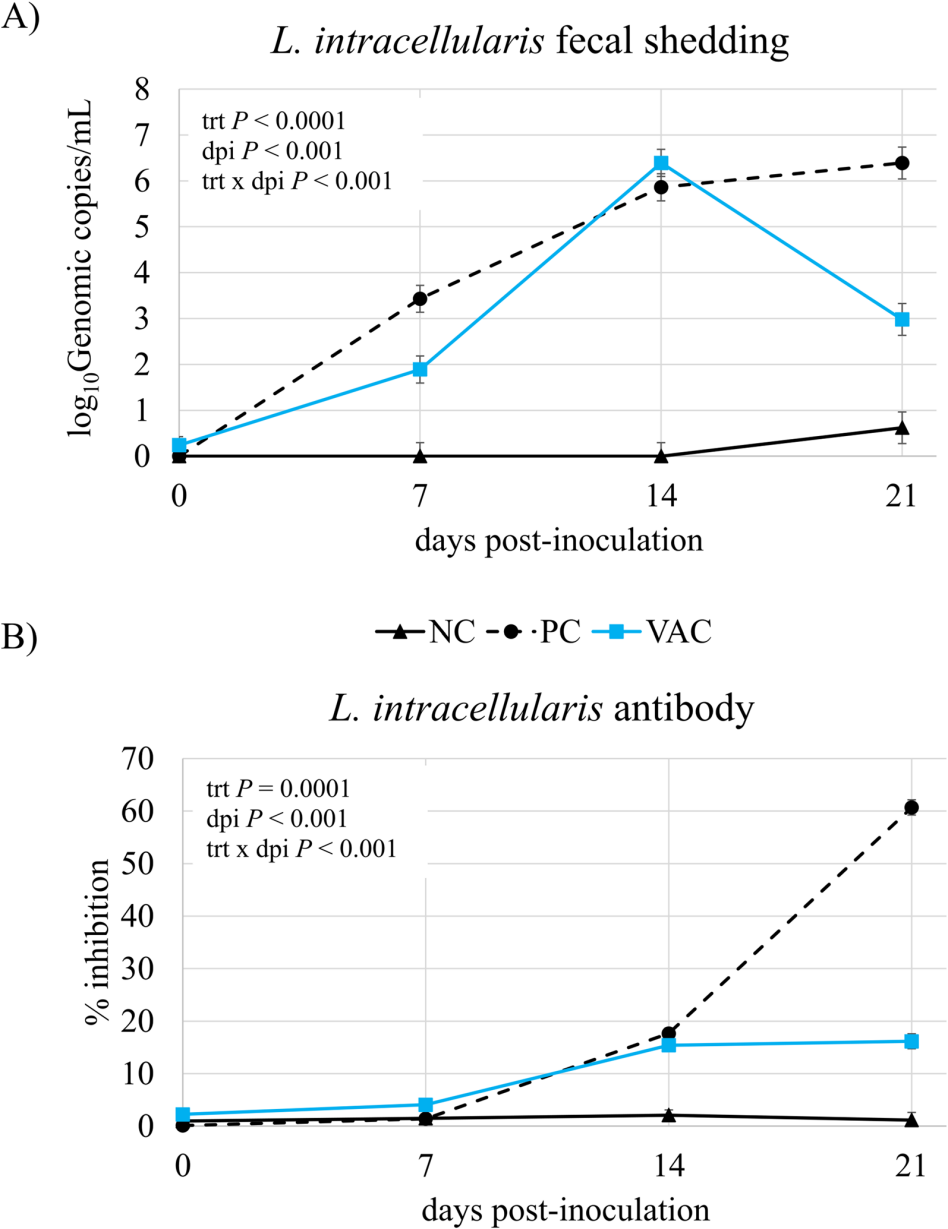
**Fecal shedding and antibody response. A** Fecal shedding and **B** antibody response in non-infected pigs (NC), non-vaccinated, *Lawsonia intracellularis* inoculated pigs (PC), and vaccinated, *Lawsonia intracellularis* inoculated pigs (VAC). PC and VAC pigs were inoculated at days post-inoculation 0 and serum and fecal swabs were collected weekly for 3 weeks. Data represents 12 pigs/treatment.

Early clinical signs of ileitis include loose stool formation, inappetence, and depression. In general, loose, formless stools and mild depression were first observed at dpi 7 and continued for the remainder of the experiment. Overall, 100% of PC pigs and 75% of VAC pigs had loose stools characteristic of enteric disease during the experimental period. Serum antibody concentrations and fecal *L. intracellularis* shedding by PCR confirmed negative status of NC pigs throughout the experimental period (Figure 1). There were three pigs in the NC group that had detectable fecal shedding at necropsy (415, 230 and 250 genomic copies/mL), this was likely either a false positive or due to contamination at necropsy, as these pigs did not test positive by any other parameters. The treatment by time interaction was significant for *L. intracellularis* fecal shedding, the (*P * <  0.001; Figure 1A). For fecal shedding, NC pigs remained negative and fecal shedding of organism continued to increase over the experimental period for PC pigs (6.4 log_10_ genomic copies/mL at dpi 21). Vaccinated pigs did shed organism after inoculation, but fecal shedding of VAC pigs peaked at dpi 14 and then began to decrease. Further, overall fecal shedding was reduced in VAC pigs compared with PC pigs (*P * <  0.001; Figure 1A).

The treatment by time interaction was significant for *L. intracellularis* antibodies (*P * <  0.001; Figure 1B), as NC pigs remained negative throughout the experiment, PC pigs had rapid induction of antibody levels, peaking at 61% inhibition at dpi 21. The serum antibody response of VAC pigs was milder, plateauing at 15–16% inhibition at dpi 14. By dpi 21, 92% of PC pigs had circulating antibody concentrations considered positive by ISU VDL thresholds (% inhibition  >  30%), while only 41% of VAC pigs were considered positive.

**Figure 2:**
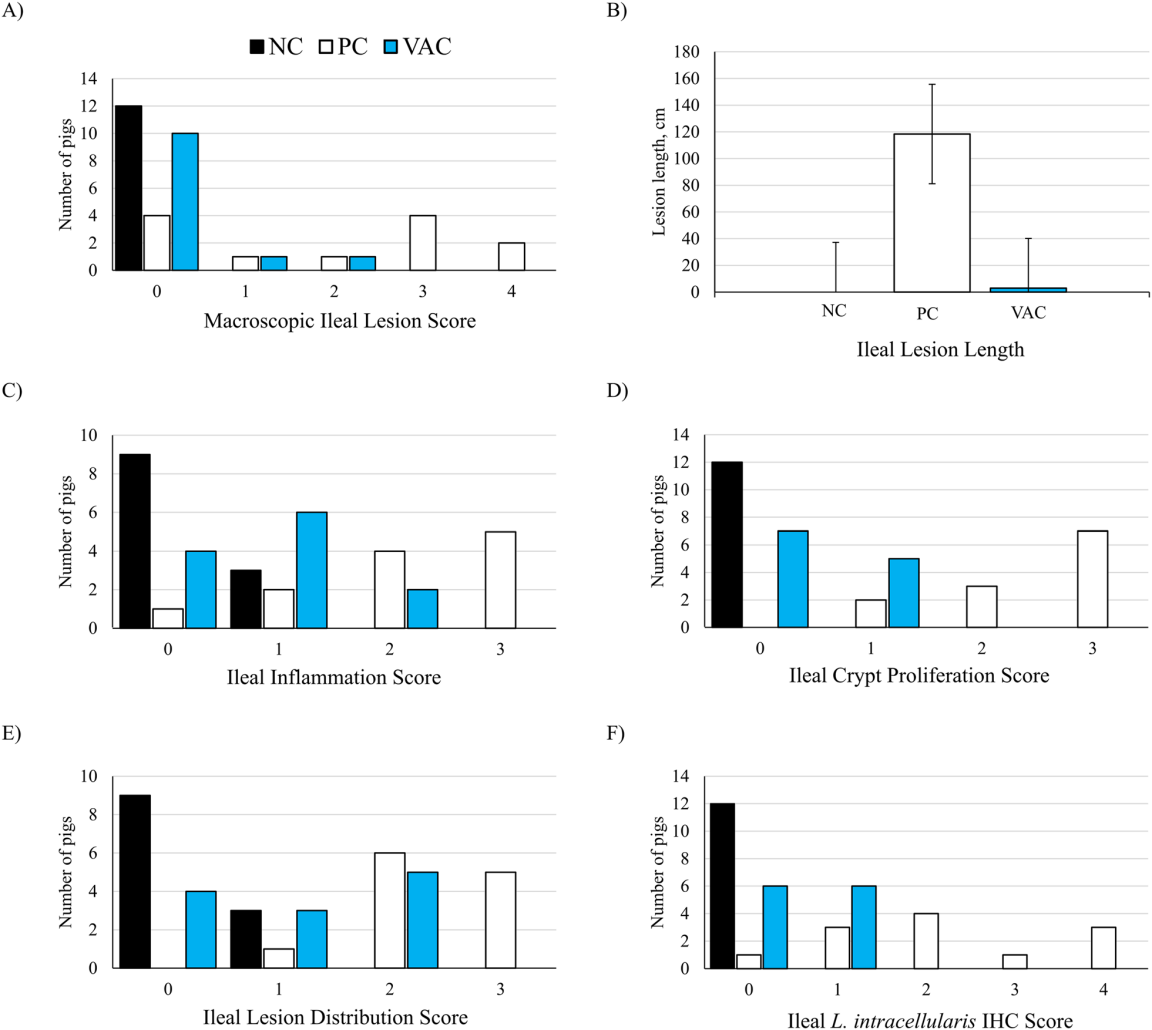
**Ileal lesion severity at days post-inoculation 21.** Panels **A** and **B** represent macroscopic lesions in non-infected pigs (NC, black), non-vaccinated *Lawsonia intracellularis* inoculated pigs (PC, white), and vaccinated *Lawsonia intracellularis* inoculated pigs (VAC, blue), while panels **C** through **F** describe microscopic lesion severity. **A** Frequency of macroscopic lesion severity score. **B** Average lesion length (cm) of lesion, if any. **C** Inflammation score wherein 0  =  none/minimal, 1  =  mild, 2  =  moderate, and 3  =  severe. **D** Proliferation of crypts, wherein 0  =  none/minimal, 1  =  mild, 2  =  moderate, and 3  =  severe. **E** Lesion distribution score, wherein 0  =  none, 1  =  focal, 2  =  multifocal, and 3  =  diffuse. **F** *L. intracellularis* immunohistochemistry (IHC) scoring, where 0  =  no antigen stain, 1  =  1–25% of crypts, 2  =  26–50% of crypts, 3  =  51–75% of crypts, 4  =   > 75% of crypts positive for antigen.

At necropsy, intestinal macroscopic and microscopic lesion scores were used to evaluate disease severity. Ileal macroscopic lesion severity was the greatest in PC pigs with 6/12 pigs scoring a three or greater. This difference was significantly greater than both VAC (*P * =  0.018) and NC (*P * =  0.003) pigs (Figure 2A). Similarly, average lesion length in the ileum was greater in PC pigs (118 cm) compared with both NC (0.0 cm, *P * =  0.007) and VAC (3.0 cm, *P * =  0.045) pigs.

Microscopic lesion scores (Figure 2) were performed in several categories encompassing degree of immune cell infiltrates (inflammation), evidence of crypt hyperplasia, distribution of lesions throughout the section, and relative IHC positive stain for *L. intracellularis*. These values were summated for an overall score for the ileum, cecum, and colon. In the ileum, all parameters differed due to treatment (Figure 2). For ileum inflammation, inflammation was greater in PC pigs compared with NC pigs (*P * <  0.001), having median inflammation scores of 2 and 0, respectively. Ileum inflammation was reduced in VAC pigs compared with PC pigs (*P * <  0.001), with VAC pigs having a median inflammation score of 1. For crypt proliferation, lesion distribution, IHC score, and overall lesion score in the ileum, all treatments significantly differed from another (*P * <  0.05 for all comparisons; Figure 2). For all these analyses, median scores for the NC pigs were 0, confirming that this group was successfully kept free of disease. The PC group had the greatest scores for these parameters, while VAC pigs had reduced score severity compared with PC pigs.

Unlike the ileum, microscopic lesion score differences were not as apparent in the large intestine (Additional file 3 and Additional file 4). In the cecum, the only difference was a tendency for a difference in IHC stain severity (*P * =  0.051), driven by a tendency for greater antigen staining in PC pigs compared with NC pigs (*P * =  0.082). In the colon, inflammation was greater in PC pigs compared with both NC pigs (*P * =  0.009) and VAC (*P * =  0.028). Crypt proliferation was greater in PC pigs compared with NC pigs only (*P * =  0.003). Lesion distribution did not differ among treatments. *Lawsonia intracellularis* IHC staining was greater in PC pigs compared with both NC (*P * =  0.003) and VAC (*P * =  0.029) pigs. Similarly, overall colon lesion severity was greater in PC pigs compared with both NC (*P * =  0.004) and VAC (*P * =  0.013) pigs.

### Growth performance

Data collected in the pre-challenge period (dpi  −7 to 0) showed there were no differences in ADG, ADFI, or G:F due to differences in room or vaccination status (Table 1). In the first week post-challenge (dpi 0–7), both ADG and ADFI differed among treatments (*P* < 0.001). Average daily gain was reduced by 33% in PC (*P * <  0.001) pigs compared with NC pigs, and did not differ between PC and VAC pigs. Similarly, ADFI was reduced in PC (27%, *P * <  0.001) pigs compared with NC pigs, and did not differ between PC and VAC pigs. Feed efficiency as assessed by Gain:Feed did not differ among treatments from dpi 0–7. From dpi 8–14, ADG was reduced 32% in PC pigs compared with NC pigs (*P * =  0.001) and did not differ between PC and VAC pigs. Feed intake was reduced 27% in PC pigs compared with NC pigs (*P * =  0.013) and did not differ between PC and VAC pigs. Feed efficiency was also reduced in PC pigs compared with both NC (*P * =  0.017) and VAC (*P * =  0.008) pigs at this time. From dpi 14–19, ADG, ADFI, and G:F were all significantly reduced in PC pigs compared with both NC and VAC pigs (*P * <  0.05 for all comparisons).

**Table 1:** **Growth performance of non-infected pigs (NC), Lawsonia intracellularis inoculated pigs (PC), and vaccinated Lawsonia intracellularis inoculated pigs (VAC)**

Treatment					
	NC	PC	VAC	SEM	*P *value
Pre-challenge, dpi-7–0					
ADG, kg/d	0.70	0.76	0.81	0.064	0.245
ADFI, kg/d	1.22	1.27	1.36	0.072	0.420
Gain:Feed	0.47	0.63	0.59	0.059	0.131
dpi 0–7					
ADG, kg/d	1.30^a^	0.87^b^	0.93^b^	0.049	< 0.001
ADFI, kg/d	2.41^a^	1.75^b^	1.99^b^	0.083	< 0.001
Gain:Feed	0.54	0.52	0.47	0.027	0.155
dpi 8–14					
ADG, kg/d	1.22^a^	0.83^b^	1.10^a^	0.070	0.001
ADFI, kg/d	2.72^a^	2.31^b^	2.40^b^	0.095	0.011
Gain:Feed	0.45^a^	0.35^b^	0.46^a^	0.025	0.005
dpi 15–19					
ADG, kg/d	1.31^a^	0.45^b^	1.03^a^	0.115	< 0.001
ADFI, kg/d	3.01^a^	2.03^b^	2.81^b^	0.169	0.001
Gain:Feed	0.46^a^	0.10^b^	0.38^a^	0.084	0.013
dpi 0–19					
ADG, kg/d	1.27^a^	0.75^c^	1.02^b^	0.044	< 0.001
ADFI, kg/d	2.68^a^	2.03^c^	2.35^b^	0.086	< 0.001
Gain:Feed	0.48^a^	0.36^b^	0.44^a^	0.017	< 0.001

Data represents 12 pigs/treatment.

dpi: days post-inoculation.

^a,b,c^Means with differing superscripts differ significantly at *P * <  0.05.

For the overall performance (dpi 0–19), ADG was reduced 41% in PC (*P * <  0.001) pigs compared with NC pigs (Table 1), while ADG was 26% greater in VAC pigs compared with PC pigs (*P * <  0.001). Similarly, ADFI was reduced in PC (24%, *P * <  0.001) pigs compared with NC pigs, and was greater in VAC pigs compared with PC pigs (14%, *P * =  0.032). Overall G:F was reduced in PC pigs compared with NC (25%; *P * <  0.001) pigs, while G:F was greater in VAC pigs compared with PC pigs (18%; *P * =  0.015; Additional file 4).

### Ex vivo* function and integrity*

In the ileum, transepithelial resistance, FD4 permeability, *S.* Typhimurium translocation, and glutamine active transport did not differ among treatments (Additional file 5). Active glucose transport in the ileum did not differ between PC and NC pigs, but tended to be greater in VAC pigs compared with PC pigs (two-fold increase; *P * =  0.074). Further, active glucose transport was significantly increased in VAC pigs compared with NC pigs (four-fold increase; *P * =  0.014). In the colon, transepithelial resistance, FD4 permeability, and *S.* Typhimurium translocation did not differ (Additional file 5).

### Intestinal morphology

Morphological parameters in the ileum, cecum, and colon are presented in Table 2. In the ileum, villus height tended to be increased in NC pigs compared with PC pigs (14% increase; *P * =  0.068) and was 20% greater in VAC pigs compared with PC pigs (*P * =  0.022). Crypt depth did not differ, thus villus:crypt ratios differed among treatments, with villus:crypt ratios being reduced in PC pigs compared with both NC (19% reduction, *P * =  0.005) and VAC (14% reduction, *P * =  0.032) pigs. In the cecum, crypt depth was greater in VAC pigs compared with NC pigs (11%, *P * =  0.031) and tended to be greater in PC pigs compared with NC pigs (10% greater, *P * =  0.083). In the colon, crypt depth was greater in PC (15% greater, *P * =  0.019) and VAC (16% greater, *P * =  0.002) pigs compared with NC pigs.

**Table 2:** **Intestinal morphology of non-infected pigs (NC), Lawsonia intracellularis inoculated pigs (PC), and vaccinated Lawsonia intracellularis inoculated pigs (VAC)**

	Treatment				
	NC	PC	VAC	SEM	*P *value
Ileum					
Villus height, μm	453^ab^	388^b^	467^a^	20.28	0.020
Crypt depth, μm	267	271	285	9.49	0.331
Villus:Crypt	1.74^a^	1.46^b^	1.67^a^	0.057	0.005
Cecum crypt depth, μm	547^b^	603^ab^	614^a^	17.92	0.026
Colon crypt depth, μm	575^b^	660^a^	685^a^	20.89	0.002

^a,b^Means with differing superscripts differ significantly at *P * <  0.05.

### Mitochondrial respiration and ROS production

**Figure 3:**
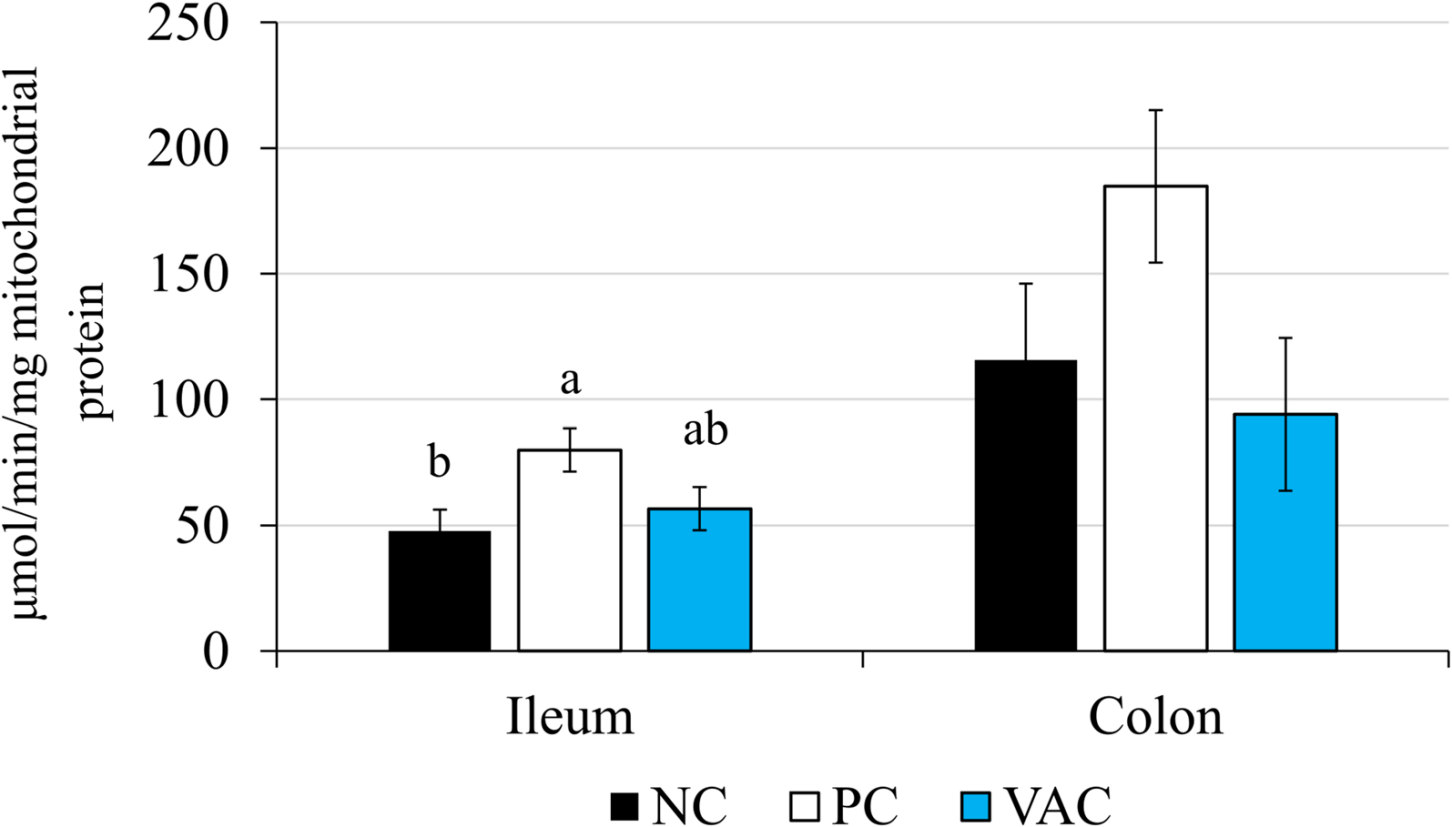
**Ileal and colonic mitochondrial reactive oxygen species (ROS) production.** ROS production was quantified in mitochondria isolated from non-infected pigs (NC), non-vaccinated *Lawsonia intracellularis* inoculated pigs (PC), and vaccinated *Lawsonia intracellularis* inoculated pigs (VAC) at days post-inoculation 21.

Mitochondrial respiration was evaluated in freshly isolated live ileal mitochondria (Additional file 6). No parameters of mitochondrial respiration differed among treatments, barring a tendency for a difference in respiratory control ratio (*P * =  0.077). This tendency was driven by increased RCR in NC pigs. Ileal mitochondrial ROS production differed among treatments, with PC pigs having 68% greater ROS production than NC pigs (*P * =  0.029; Figure 3). Colon ROS production had similar numerical trends, but these did not reach significance (*P * =  0.103; Figure 3).

### Ileum cytokine concentrations

Ileum cytokine concentrations are presented in Table 3. Concentrations of interleukin (IL)—1α tended to be greater in PC pigs compared with NC pigs (24-fold increase, *P * =  0.056) and tended to be reduced in VAC pigs compared with PC pigs (21-fold reduction, *P * =  0.058). Concentrations of IL-1β were increased 13-fold in PC pigs compared with NC pigs (*P * =  0.015) and were reduced 17-fold in VAC pigs compared with PC pigs (*P * =  0.013). Concentrations of IL-1ra were increased three-fold in PC pigs compared with NC pigs (*P * =  0.002) and were reduced three-fold in VAC pigs compared with PC pigs *(P * =  0.002). Concentrations of IL-2 were 60% greater in PC pigs compared with NC pigs (*P * =  0.021). Concentrations of IL-6 tended to be greater in PC pigs compared with NC pigs (six-fold increase, *P * =  0.073). Concentrations of IL-10 were three-fold greater in PC pigs compared with NC pigs (*P * <  0.001) and were reduced two-fold in VAC pigs compared with PC pigs (*P * =  0.002). Concentrations of IL-18 tended to differ among treatments (*P * =  0.071). Concentrations of IL-4, IL-8, and IL12 did not differ among treatments.

**Table 3:** **Ileal cytokine concentrations in non-infected pigs (NC), Lawsonia intracellularis inoculated pigs (PC), and vaccinated Lawsonia intracellularis inoculated pigs (VAC)**

Item^c^	Treatment			SEM	*P* value
	NC	PC	VAC		
IL-1α	0.45^y^	10.79^x^	0.52^y^	3.046	0.032
IL-1β	14.17^b^	192.63^a^	11.07^b^	42.50	0.006
IL-1ra	2.83^b^	7.68^a^	2.93^b^	0.901	0.001
IL-2	0.31^b^	0.50^a^	0.39^ab^	0.049	0.028
IL-4	0.09	0.18	0.24	0.072	0.32
IL-6	0.25	1.44	0.37	0.369	0.057
IL-8	91.52	82.49	86.11	9.931	0.812
IL-10	0.07^b^	0.21^a^	0.10^b^	0.021	< 0.001
IL-12	2.22	1.85	1.84	0.343	0.673
IL-18	161.8	130.1	115.6	13.98	0.071

^a,b^Means with differing superscripts differ significantly at *P * <  0.05.

^x,y^Means with differing superscripts differ significantly at *P * <  0.10.

^c^pg/mg isolated protein.

### Digestibility

Apparent ileal digestibility coefficients did not differ among treatments (Table 4; *P * >  0.10), however high variability was associated with these measures. For apparent cecal digestibility, all coefficients differed among treatments. Dry matter ACD was reduced 17% in PC pigs compared with NC pigs (*P * <  0.001) and was 13% greater in VAC pigs compared with NC pigs (*P * <  0.001). Nitrogen ACD was reduced 13% in PC and VAC pigs compared with NC pigs (*P * <  0.001) and did not differ between PC and VAC pigs. Organic matter ACD was reduced 16% in PC pigs compared with NC pigs (*P * <  0.001) and was 11% greater in VAC pigs compared with PC pigs (*P * =  0.004). Similarly, GE ACD was reduced 17% in PC pigs compared with NC pigs (*P * <  0.001) and was 14% greater in VAC pigs compared with PC pigs (*P * =  0.004).

**Table 4:** **Apparent ileal, cecal, and total tract digestibility coefficients of dry matter (DM), nitrogen (N), organic matter (OM), and energy (GE) in non-infected pigs (NC), Lawsonia intracellularis inoculated pigs (PC), and vaccinated Lawsonia intracellularis inoculated pigs (VAC)**

Treatment					
	NC	PC	VAC	SEM	*P* value
Apparent ileal digestibility					
DM	53.4	43.9	56.7	10.51	0.659
N	68.9	17.3	59.1	24.59	0.282
OM	56.4	48.2	60.2	9.86	0.664
GE	54.9	42.4	59.3	11.70	0.553
Apparent cecal digestibility					
DM	72.3^a^	59.9^b^	69.0^a^	1.66	< 0.001
N	72.4^a^	62.9^b^	65.9^b^	1.39	< 0.001
OM	74.5^a^	62.6^b^	70.7^a^	1.63	< 0.001
GE	72.6^a^	60.4^b^	68.7^a^	1.67	< 0.001
Apparent total tract digestibility					
DM	83.4^a^	80.2^b^	81.5^ab^	0.54	0.003
N	80.3^a^	73.9^b^	76.3^b^	1.07	0.001
OM	84.9^a^	82.3^b^	83.6^ab^	0.52	0.006
GE	82.4^a^	79.3^b^	80.8^ab^	0.58	0.002

Data represents 12 pigs/treatment.

^a,b^Means with differing superscripts differ significantly at *P * <  0.05.

Apparent total tract digestibility coefficients were also affected by treatment (Table 4). Dry matter ATTD was reduced 3% in PC pigs compared with NC pigs (*P * =  0.002). Nitrogen digestibility was reduced 8% in PC pigs compared with NC pigs (*P * =  0.001). Organic matter ATTD was reduced 3% in PC pigs compared with NC pigs (*P * =  0.004). Similarly, GE ATTD was reduced 4% in PC pigs compared with NC pigs (*P * =  0.001). For all these parameters, ATTD values did not differ significantly between VAC and PC pigs, however numerical increases were observed (1.5%, 3%, 1.6%, and 1.8% increase in DM, N, OM, and GE ATTD, respectively).

### Ileal gene abundance

**Figure 4:**
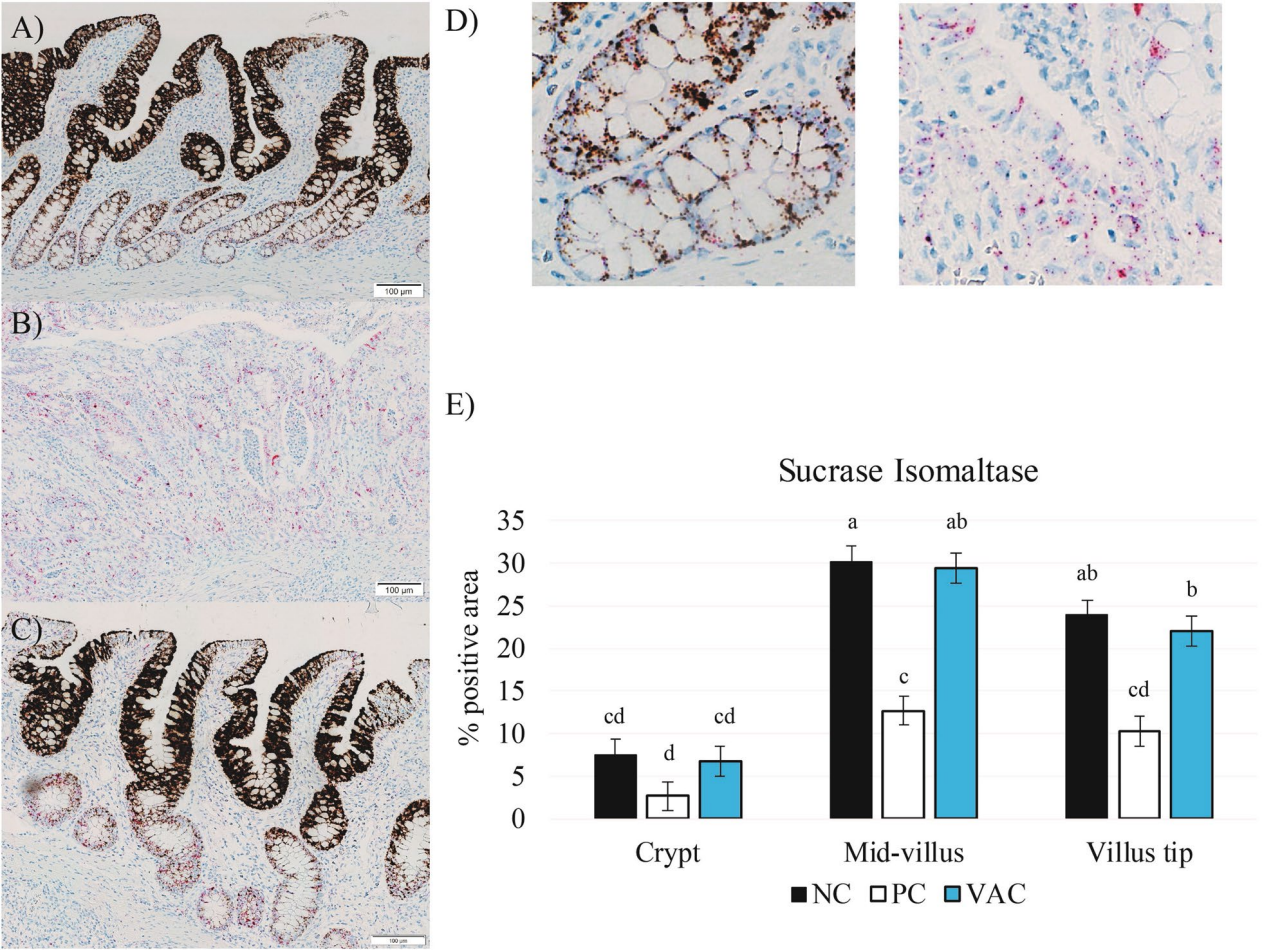
**RNA chromogenic in-situ hybridization dual probe for sucrase-isomaltase (brown) and Hes1 (red).** Transcripts were quantified in the ileum of non-infected pigs (NC), non-vaccinated *Lawsonia intracellularis* inoculated pigs (PC), and vaccinated *Lawsonia intracellularis* inoculated pigs (VAC) at days post-inoculation 21. Representative images of dual stain in **A** NC, **B** PC, and **C** VAC pigs. Sucrase-isomaltase transcript was found throughout the intestinal epithelium, particularly concentrated in the mid-villus region and villus tips. Near complete abolition of this transcript was observed in affected crypts in pigs challenged with *L. intracellularis*. The abundance of transcription factor Hes1 was also evaluated by RNA in-situ hybridization. **D** Example of Hes1 staining in the crypts. Hes1 transcript appeared sporadically throughout the crypt-villus axis in all treatment groups. However, due to the overwhelming sucrase-isomaltase staining, this transcript was unable to be quantified. **E** Quantification of sucrase isomaltase stain in the crypt, mid-villus, and villus tip.

To investigate mechanisms by which *L. intracellularis* challenge may reduce growth performance and digestive function, sucrase-isomaltase (SI) mRNA abundance was quantified in the ileal epithelium (Figure 4). In general, SI mRNA was found throughout the intestinal epithelium, particularly concentrated in the mid-villus region and villus tips. However, near complete abolition of this transcript was observed in affected crypts of PC pigs (Figure 4). Overall, PC pigs had reduced SI transcript compared with both NC and VAC pigs at all regions of interest along the villus-crypt axis (*P * <  0.01 for all), this difference being greatest in the mid-villus region. The abundance of transcription factor Hes1, a component of the Notch signaling pathway indicative of cells predestined to become absorptive enterocytes [14], was also evaluated by RNA in-situ hybridization. However, due to the overwhelming transcript intensity of SI, it was not possible to confidently quantify abundance of this transcript. In general, Hes1 transcript appeared sporadically throughout the crypt-villus axis in all treatment groups. Although visually PC pigs appeared to have greater abundance of Hes1 transcript, this could have been due to the lack of SI transcript, which likely obfuscates Hes1 transcript in the other treatment groups.

Additionally, mRNA abundance of several markers of epithelial turnover and maturity were evaluated by PCR (Table 5). Abundance of ATOH1 and β-catenin did not differ among treatments. WNT3A mRNA abundance was two-fold greater in PC pigs compared with NC pigs (*P * =  0.046) and two-fold greater in PC pigs compared with VAC pigs (*P * =  0.039). Transcript abundance of Hes1 was two-fold greater in PC pigs compared with NC pigs (*P * =  0.039) and did not differ between VAC and PC pigs. Relative abundance of p27^Kip1^ did not differ between NC and PC pigs but tended to be greater in PC pigs compared with VAC pigs (*P * =  0.074). Relative abundance of IAP was lesser in PC pigs (*P * =  0.003) compared with NC pigs and did not differ between PC and VAC pigs. Abundance of SI was two-fold lesser in PC pigs compared with NC pigs (*P * =  0.047), however did not differ significantly between PC and VAC pigs. Similarly, relative abundance of MUC2 was lower in PC pigs compared with NC pigs (*P * =  0.037). Abundance of SI and MUC2 did not differ significantly between PC and VAC pigs.

**Table 5:** **Ileal mRNA abundance in non-infected pigs (NC), Lawsonia intracellularis inoculated pigs (PC), and vaccinated Lawsonia intracellularis inoculated pigs (VAC)**

Treatment					
Gene name	NC	PC	VAC	SEM	*P* value
ATOH1	1.10	1.14	1.36	0.181	0.558
β-catenin	1.20	1.37	0.99	0.148	0.187
WNT3A	0.98^b^	1.98^a^	0.95^b^	0.291	0.022
Hes1	1.00^b^	1.81^a^	1.82^a^	0.221	0.019
p27kip1	1.10	1.62	0.83	0.250	0.085
IAP	1.34^a^	0.33^b^	0.65^b^	0.196	0.003
SI	1.27^a^	0.61^b^	0.69^ab^	0.189	0.047
MUC2	1.22^a^	0.52^b^	0.94^ab^	0.205	0.046

Data represents 12 pigs/treatment.

^a,b^Means with differing superscripts differ significantly at *P * <  0.05.

## Discussion

*Lawsonia intracellularis* is an obligate intracellular pathogen responsible for ileitis, a major enteric disease for pigs in the grower and early finishing periods [1]. Global herd prevalence of the pathogen approaches 96%, and it is estimated that approximately 30% of grower and finisher pigs will be afflicted with *L. intracellularis* at some point in their production lives [2, 3]. However, much remains unknown regarding its impact on intestinal physiology. Gene abundance studies [8, 9, 26] have observed mRNA transcript changes to pathways involved in cellular transport, inflammation, and mucus production during peak infection which would suggest *L. intracellularis* reduces nutrient digestion, nutrient transport, and mucosal integrity. Further, the pathogen is purported to induce inflammatory and cell proliferation pathways associated with disease [26]. However, the functional implications this pathogen and its associated disease has on digestibility and intestinal permeability are largely unknown. Therefore, this study aimed to evaluate intestinal function and integrity in *L. intracellularis* challenged pigs, and the ability of a live, attenuated vaccine to impact these parameters.

Field evaluation of *L. intracellularis* vaccination programs in endemically afflicted herds report that vaccinated pigs have reduced incidence and severity of ileitis and greater body weight gains compared with non-vaccinated pigs [27–29]. However, the magnitude of growth and feed efficiency improvement in more controlled experimentally challenged pigs is less clear. Kroll et al. [30] reported that vaccinated pigs had greater growth rates than nonvaccinated pigs following experimental challenge, although these pigs were only weighed at dpi 0 and 21 and feed efficiency was not evaluated. In the current study, we evaluated performance parameters every 7 days throughout the 21 days challenge period. We observed vaccinated pigs to have the same initial reduction in growth following the experimental challenge compared to non-vaccinates, but vaccinated pigs had greater gains than their non-vaccinated counterparts for the remaining 14 days of experimental challenge. Overall, vaccinated pigs had a 26% improvement in growth compared with PC pigs, indicating significant protection from the performance impacts of disease. Further, the reduction in feed efficiency observed in non-vaccinated pigs was not observed in vaccinated pigs. These data suggest that vaccination prevents disease associated losses in feed efficiency and reductions in growth were largely due to reduced feed intake [13, 31].

We also observed vaccinated pigs to have reduced fecal shedding, serum antibody response, and lesions associated with *L. intracellularis* infection, consistent with previous reports [30, 32]. Oral live attenuated vaccines induce innate and adaptive immune responses at the mucosa, resulting in antigen specific antibody populations which are quickly mobilized to neutralize bacteria [33, 34]. Thus, it is likely vaccinated pigs were still susceptible to the initial challenge, but were able to quickly mobilize a memory immune response to clear the pathogen and recover, improving growth rates in the latter part of the study and reducing intestinal lesions at dpi 21. Taken together, vaccination mitigated disease and improved growth of *L. intracellularis* experimentally challenged pigs; thus, we then aimed to evaluate if disease mitigation was associated with changes to intestinal physiology.

Maintaining barrier integrity during pathogen challenge is essential to prevent further dysbiosis and secondary infection. Critical to this barrier are mucus production by goblet cells and the tight junctions between epithelial cells themselves. *Lawsonia intracellularis* causes goblet cell depletion and reduces production of mucins [the current study; 9, 10, 35], which may increase intestinal permeability via mucus layer depletion. However, microarray studies of *L. intracellularis* infected tissues have found little evidence of downregulation in tight junction components. Smith et al. [9] only found reductions in the abundance of 2 tight junction associated protein mRNA transcripts, claudin-15 and HEPACAM2 (HEPACAM family member 2). However, claudin-15 functions primarily as a pore forming protein [36], and HEPACAM2 is a purported tumor suppressor [37], thus reductions to their abundance likely would not increase epithelial permeability. Functionally, *L. intracellularis/Mycoplasma hyopneumoniae* co-challenged pigs were found to have increased ex vivo colonic permeability to *S.* Typhimurium, however no changes to transepithelial resistance [38]. In the current study, we observed no change to transepithelial resistance, macromolecule permeability (FD4), or ex vivo *S.* Typhimurium translocation in the ileum or colon. Rather than entering the pig via paracellular mechanisms, *Salmonella* Typhimurium penetrates the epithelium transcellularly by hijacking host macropinocytosis mechanisms [39–41]. *L. intracellularis* seroconversion has been shown to increase *Salmonella* shedding [42] and vaccination temporally decreases the shedding of *S.* Typhimurium in co-challenged pigs [43]. The finding that *Salmonella* translocation ex vivo was not impacted by *L. intracellularis* challenge or vaccination suggests that the mechanisms involved in the interactions among these two pathogens are likely not associated with changes in intestinal permeability but rather changes in microbiome composition and/or immune responses [43, 44].

Intestinal immune responses during *L. intracellularis* infection includes large numbers of infiltrating macrophages, which are found throughout the ileal lamina propria at peak infection [9]. Further, increased cleaved-caspase-3 has been observed at infected crypt lumens during peak infection, consistent with macrophage-induced apoptosis [35]. Macrophages degrade phagocytosed bacteria and induce lumen-associated apoptosis of infected cells by oxidative burst, partially driven by enhanced mitochondrial ROS generation [45]. Thus, it is likely a greater number of macrophages present in PC pig ileal tissue led to the greater mitochondrial ROS production rates reported herein. Consistent with this postulation, previous work has found increased abundance of xanthine dehydrogenase in *L. intracellularis* challenged pigs, an enzyme which regulates production of ROS and nitric oxide synthase to control bacterial infections [26].

In their response to intracellular pathogens, macrophages produce a myriad of cytokines, including IL-1β, IL-6, IL-12, and IL-18 [46], which promote recruitment of other immune components. In the current experiment, we observed increased concentrations of several proinflammatory cytokines, most notably IL-1β. In addition to production by macrophages, IL-1β production by infected enterocytes is critical in the formation of the inflammasome, a key component of controlling and clearing intracellular pathogens [47, 48]. However, excessive production of this cytokine could be responsible for a number of effects of pig metabolism, including reduced feed intake [49], impaired nutrient transporter function [50], or even hyperplasia of intestinal enterocytes [26, 51]. The vaccinated pigs had lesser evidence of inflammation, as many proinflammatory cytokine concentrations were equivalent to that of the NC control pigs, which likely contributed to their greater performance and health during pathogen challenge.

As a hallmark of *L. intracellularis* infection is extensive proliferation and hyperplasia of undifferentiated or immature enterocytes [1, 52] it is likely that reduced digestion and malabsorption contribute to the reduced growth associated with ileitis. Hamsters infected with *L. intracellularis* have impaired intestinal glucose absorption, further suggesting impaired digestibility [7]. However, digestibility of nutrients during *L. intracellularis* challenge in pigs has been poorly characterized. Visscher et al. [53] observed no differences in AID of nutrients between clinically or non-clinically afflicted during a natural *L. intracellularis* challenge. These researchers did observe a reduction in crude protein ATTD in non-vaccinated, clinically afflicted pigs compared with non-vaccinated, non-clinically afflicted pigs and vaccinated pigs, but no differences in other ATTD parameters (i.e., N, DM) between vaccination or ileitis disease states [53]. However, this study did not include any true negative control pigs, so it is difficult to draw conclusions regarding the impact of *L. intracellularis* alone on nutrient digestibility. To our knowledge, the study herein is the first report comparing digestibility of *L. intracellularis* challenged pigs (PC) with NC pigs. In the current experiment, no significant changes to AID were observed. However, these samples were collected at a single timepoint (necropsy) so high animal-to-animal variation was observed. Sloughing of cells during necropsy can affect N and amino acid digestibility numbers, contributing to variation [54]. Further, PC pigs had high variation in feed intake and digesta viscosity which further compounded sample variation, suggesting evaluating AID via the slaughter method may not be a particularly useful metric during digestive disorders such as *L. intracellularis* challenge. Numerically, PC pigs had reductions in ileal digestibility, particularly nitrogen, which was most apparent in pigs clinically affected at the time of necropsy. This is possibly a consequence of increased cell sloughing or reduced reabsorption of endogenous nitrogen, which primarily occurs at the distal end of the small intestine [55].

Converse to AID, reductions in both ACD and ATTD were observed in PC pigs compared with NC pigs. Vaccination was able to either partially (total tract) or fully (cecal) mitigate these losses for all parameters barring nitrogen digestibility. Reduced ATTD has been observed due to other enteric diseases including porcine epidemic diarrhea virus [22] and *Brachyspira hyodysenteriae* [56], as well as resulting from lipopolysaccharide inflammatory [57] and porcine reproductive and respiratory syndrome virus challenges [58]. However, the mechanisms responsible for reduced nutrient digestibility likely vary due to each stressor, age of pig, digesta flow, and other environmental factors. In the case of *L. intracellularis* challenge, it is likely the failure of cells to fully differentiate leads to reduced abundance of digestive enzymes and transporters. Indeed, *L. intracellularis* challenged hamsters have impaired nutrient absorption [7], and challenged pigs have reduced activity of ileal sucrase [38] and reduced ileal mRNA abundance of nutrient transporters [8, 9]. In the current experiment PC pigs had near complete abolition of the mRNA transcript for the brush border glucosidase sucrase-isomaltase (SI) measured via RNAscope, a finding confirmed by PCR on ileal mucosal scrapings. Interestingly, VAC pigs had SI transcript abundance similar to that of NC pigs, indicating enhanced maturity and absorptive function that likely contributed to improved digestibility and growth during the challenge. However, when evaluated via PCR, VAC pigs did not have a significant increase in SI abundance compared with PC pigs. This discrepancy may be because *L. intracellularis* does not affect the entire ileum equally or due to differences between the techniques. The PC pigs also had reduced abundance of the mRNA transcript for *IAP*, a protective enzyme often used as a marker of enterocyte maturity [59], further supporting reduced maturity and absorptive capacity in *L. intracellularis* infected tissues. However, upstream signaling surrounding this phenomenon has not fully been elucidated.

It is thought that *L. intracellularis* induces a greater proliferation rate of progenitor cells, a population of pre-differentiated epithelial cells [35]. Under normal circumstances, the epithelial layer is maintained by a population of intestinal stem cells (ISCs) that reside at the base of the crypts. These cells give rise to daughter progenitor cells that rapidly proliferate and migrate out of the crypts. As they migrate, progenitor cells cease proliferation and begin to differentiate into the various intestinal cell lineages: neuroendocrine cells, Paneth cells, goblet cells, or absorptive enterocytes [60]. However, as *L. intracellularis* infected epithelial cells lack capacity for mucin secretion and have attenuated digestive and absorptive capacity, we hypothesized that some aberrant signaling prevents their full differentiation and maturation. Two of the major signaling pathways responsible for ISC differentiation and maturation are the canonical β-catenin/Wnt and Notch signaling pathways [61]. Generally speaking, the canonical β-catenin/Wnt pathway is necessary for maintenance of proliferative stem cells at the crypt [61], and differentiation into Paneth cells. When Wnt is not present, cytosolic β-catenin is constantly degraded by the adenomatous polyposis coli and Axin complexes, which prevents β-catenin from reaching the nucleus and acting as a transcriptional coactivator of Wnt target genes [62]. Conversely, presence of Wnt prevents degradation and allows cytosolic accumulation and nuclear translocation of β-catenin, leading to Wnt target gene expression [62]. This signaling pathway is highly active in cells at the base of the crypts, and decreases as cells move up the crypt-villus axis and begin the differentiation process [63]. Interestingly, over activation of the β-catenin/Wnt signaling pathway can cause epithelial cells to enter a proliferative state with a failure to differentiate, and this is often observed in hyperproliferative conditions including gastrointestinal cancers [63–66]. In the current experiment, we observed a two-fold increase of the WNT3A transcript in PC pigs at dpi 21. While no significant change to β-catenin transcript abundance was observed, this transcript was numerically increased. Similarly, Huan et al. [35] observed increased cytosolic β-catenin in *L. intracellularis* infected epithelial cells at dpi 7 and 14, which corresponded to peak infection. Contradictory to our data herein, these researchers also observed a concurrent reduction in WNT3A transcript abundance and postulated that *L. intracellularis* may downregulate β-catenin/Wnt signaling at the peak of infection [35]. However, Leite et al. [26] did observe activation of the Wnt/Ca^+^ pathway in *L. intracellularis* challenged pigs. Disparities in WNT3A transcript abundance between these studies may be due to differences in disease severity and progression. Regardless, results of all three studies suggest that over activation of β-catenin/Wnt could keep *L. intracellularis* infected epithelial cells in a pre-differentiated or immature state.

As epithelial cells differentiate, the Notch signaling pathway is critical in determining their fate as either an absorptive enterocyte or goblet cell precursor. Notch signaling in progenitor cells enhances the expression of components of the hairy enhancer of split (Hes) complex (Hes1-Hes7 and Hey1-Hey3, a helix-loop-helix transcriptional repressor) which promotes differentiation of progenitor cells into absorptive cells [14]. Concurrently, Hes1 represses transcription of atonal homolog 1 (ATOH1), a promotor of differentiation towards the secretory lineage [14]. In the current experiment, we observed increased mRNA abundance of Hes1 in PC pigs with no change to the abundance of ATOH1 compared with NC pigs at dpi 21. Others have reported upregulation of the Notch-1 receptor in *L. intracellularis* infected crypts at peak infection [35], supporting active Notch signaling in *L. intracellularis* challenged pigs. In addition to controlling epithelial cell fates, Notch signaling is also partially responsible for maintaining cells in the proliferative progenitor state. Although the exact mechanisms are unclear, it appears likely that this is either via Hes1-induced repression of cyclin-dependent kinase inhibitors p27^Kip1^ and p57^kip2^ [67] or through ATOH1 repression [68]. Although PC pigs did not have reduced mRNA abundance of either p27^Kip1^ or ATOH1, there are many partial or full redundancies in the Notch signaling pathway, so lack of reductions in their abundance does not necessarily contradict augmented Notch signaling [61]. Regardless, these results suggest the hyperplasia induced by *L. intracellularis* in the current study may be partially driven by heightened activity of both β-catenin/Wnt and Notch signaling pathways, which induce proliferation while preventing cells from developing functional capacity. Similarly, Wnt and Notch pathways have been shown to synergistically contribute to hyperplasia in gastrointestinal tumorigenesis [69], a model which also involves excessive proliferation of undifferentiated epithelial cells. Previous researchers have made connections between *L. intracellularis* induced proliferation and tumorigenesis, with a transcriptomic study of *L. intracellularis* challenged pigs [26]. These authors associated host-driven inflammation with upregulation of several proliferative pathways found in tumor cells, including transglutaminase-2 and oncostatin M, a member of the IL-6 family [26]. In further support of this result, the current experiment observed significant intestinal inflammation in PC pigs, including a six-fold increase in IL-6. This link is unsurprising, as both pathologies involve uncontrolled and excessive cellular proliferation. However, in the case of *L. intracellularis* infection, the immune system eventually clears the bacteria to resolve proliferative lesions and restore digestive function.

Taken together, the results of this study demonstrate that *L. intracellularis* associated reductions in growth performance can be partially attributed to a reduction in digestibility, likely driven by abolition of absorptive and digestive enzymes in infected epithelial cells at peak infection. Additionally, *L. intracellularis* induces intestinal inflammation and epithelial hyperplasia in a similar manner to some intestinal cancers in accordance with previous findings [26], involving activation of β-catenin/Wnt and Notch signaling pathways to maintain cells in a proliferative progenitor state [35]. Further, this study demonstrates that vaccination for *L. intracellularis* significantly improves pig performance and reduces lesion severity in pigs after experimental challenge, likely driven by reduced pathogen load which reduced ileal inflammation.

## Supplementary Information

**Additional file 1** **Diet composition, as fed.**

**Additional file 2** **Primer sequences.**

**Additional file 3** **Macroscopic lesion severity.**

**Additional file 4** **Microscopic lesion severity.**

**Additional file 5** **Ex vivo intestinal integrity and function parameters.**

**Additional file 6** **Ileal and colonic mitochondrial parameters.**

## Data Availability

All relevant data are within the manuscript and its Additional Information files.

## Abbreviations

ACD: Apparent cecal digestibility
ADFI: Average daily feed intake
ADG: Average daily gain
AID: Apparent ileal digestibility
ATTD: Apparent total tract digestibility
ATOH1: Atonal homolog 1
CA: Bicinchoninic acid
DCFH: 2′,7′-Dichlorofluorescin diacetate
DM: Dry matter
dpi: Days post-inoculation
FD4: Fluorescein isothiocyanate-dextran 4 kDa
GE: Gross energy
G:F: Gain:Feed
Hes: Hairy enhancer of split
IAP: Intestinal alkaline phosphatase
IL: Interleukin
ISCs: Intestinal stem cells
ISU VDL: Iowa State University Veterinary Diagnostic Lab
N: Nitrogen
OM: Organic matter
ROS: Reactive oxygen species
SI: Sucrase-isomaltase

## References

[1] Vannucci FA, Gebhart CJ, McOrist S, Zimmerman JJ, Karriker LA, Ramirez A, Schwartz KJ, Stevenson GW (2019). Proliferative enteropathy. Diseases of swine.

[2] Stege H, Jensen TK, Moller K, Baekbo P, Jorsal SE (2000). Prevalence of intestinal pathogens in Danish finishing pig herds. Prev Vet Med.

[3] McOrist S, Barcellos D, Wilson R (2003). Global patterns of porcine proliferative enteropathy. Pig J.

[4] Paradis MA, Gebhart CJ, Toole D, Vessie G, Winkelman NL, Bauer SA, Wilson JB, McClure CA (2012). Subclinical ileitis: diagnostic and performance parameters in a multi-dose mucosal homogenate challenge model. J Swine Health Prod.

[5] Guedes RMC, Machuca MA, Quiroga MA, Pereira CER, Resende TP, Gebhart CJ (2017). *Lawsonia intracellularis* in pigs: progression of lesions and involvement of apoptosis. Vet Pathol.

[6] Lawson GH, Gebhart CJ (2000). Proliferative enteropathy. J Comp Pathol.

[7] Vannucci FA, Borges EL, de Oliveira JS, Guedes RM (2010). Intestinal absorption and histomorphometry of Syrian hamsters (*Mesocricetus auratus*) experimentally infected with *Lawsonia intracellularis*. Vet Microbiol.

[8] Vannucci FA, Foster DN, Gebhart CJ (2013). Laser microdissection coupled with RNA-seq analysis of porcine enterocytes infected with an obligate intracellular pathogen (*Lawsonia intracellularis*). BMC Genomics.

[9] Smith SH, Wilson AD, Van Ettinger I, MacIntyre N, Archibald AL, Ait-Ali T (2014). Down-regulation of mechanisms involved in cell transport and maintenance of mucosal integrity in pigs infected with *Lawsonia intracellularis*. Vet Res.

[10] Bengtsson RJ, MacIntyre N, Guthrie J, Wilson AD, Finlayson H, Matika O, Pong-Wong R, Smith SH, Archibald AL, Ait-Ali T (2015). *Lawsonia intracellularis* infection of intestinal crypt cells is associated with specific depletion of secreted MUC2 in goblet cells. Vet Immunol Immunopathol.

[11] Peiponen KS, Tirkkonen BT, Junnila JJT, Heinonen ML (2018). Effect of a live attenuated vaccine against *Lawsonia intracellularis* in weaned and finishing pig settings in Finland. Acta Vet Scand.

[12] National Research Council (2012). Nutrient requirements of swine.

[13] Helm ET, Curry SM, De Mille CM, Schweer WP, Burrough ER, Gabler NK (2020). Impact of viral disease hypophagia on pig jejunal function and integrity. PLoS One.

[14] Kazanjian A, Shroyer NF (2011). NOTCH signaling and ATOH1 in colorectal cancers. Curr Colorectal Cancer Rep.

[15] Helm ET, Curry S, Trachsel JM, Schroyen M, Gabler NK (2019). Evaluating nursery pig responses to in-feed sub-therapeutic antibiotics. PLoS One.

[16] Helm ET, Lin SJ, Gabler NK, Burrough ER (2020). *Brachyspira hyodysenteriae* infection reduces digestive function but not intestinal integrity in growing pigs while disease onset can be mitigated by reducing insoluble fiber. Front Vet Sci.

[17] Iqbal M, Pumford NR, Tang ZX, Lassiter K, Wing T, Cooper M, Bottje W (2004). Low feed efficient broilers within a single genetic line exhibit higher oxidative stress and protein expression in breast muscle with lower mitochondrial complex activity. Poult Sci.

[18] Grubbs JK, Fritchen AN, Huff-Lonergan E, Dekkers JC, Gabler NK, Lonergan SM (2013). Divergent genetic selection for residual feed intake impacts mitochondria reactive oxygen species production in pigs. J Anim Sci.

[19] Helm ET, Lin SJ, Gabler NK, Burrough ER (2020). *Brachyspira hyodysenteriae* infection reduces digestive function but not intestinal integrity in growing pigs while disease onset can be mitigated by reducing insoluble fiber. Front Vet Sci.

[20] Rogers GW, Brand MD, Petrosyan S, Ashok D, Elorza AA, Ferrick DA, Murphy AN (2011). High throughput microplate respiratory measurements using minimal quantities of isolated mitochondria. PLoS One.

[21] Luso A, Repp B, Biagosch C, Terrile C, Prokisch H (2017). Assessing mitochondrial bioenergetics in isolated mitochondria from various mouse tissues using seahorse XF96 analyzer. Methods Mol Biol.

[22] Schweer WP, Schwartz K, Burrough ER, Yoon KJ, Sparks JC, Gabler NK (2016). The effect of porcine reproductive and respiratory syndrome virus and porcine epidemic diarrhea virus challenge on growing pigs I: growth performance and digestibility. J Anim Sci.

[23] Leone JL (1973). Collaborative study of the quantitative determination of titanium dioxide in cheese. J Assoc Off Anal Chem.

[24] Faithfull NT (2002). Methods in agricultural chemical analysis: a practical handbook.

[25] Oresanya TF, Beaulieu AD, Patience JF (2008). Investigations of energy metabolism in weanling barrows: the interaction of dietary energy concentration and daily feed (energy) intake. J Anim Sci.

[26] Leite FL, Abrahante JE, Vasquez E, Vannucci F, Gebhart CJ, Winkelman N, Mueller A, Torrison J, Rambo Z, Isaacson RE (2019). A cell proliferation and inflammatory signature is induced by *Lawsonia intracellularis* infection in swine. MBio.

[27] Park S, Lee JB, Kim KJ, Oh YS, Kim MO, Oh YR, Hwang MA, Lee JA, Lee SW (2013). Efficacy of a commercial live attenuated *Lawsonia intracellularis* vaccine in a large scale field trial in Korea. Clin Exp Vaccine Res.

[28] McOrist S, Smits RJ (2007). Field evaluation of an oral attenuated *Lawsonia intracellularis* vaccine for porcine proliferative enteropathy (ileitis). Vet Rec.

[29] Almond PK, Bilkei G (2006). Effects of oral vaccination against *Lawsonia intracellularis* on growing-finishing pig’s performance in a pig production unit with endemic porcine proliferative enteropathy (PPE). Dtsch Tierarztl Wochenschr.

[30] Kroll JJ, Roof MB, McOrist S (2004). Evaluation of protective immunity in pigs following oral administration of an avirulent live vaccine of *Lawsonia intracellularis*. Am J Vet Res.

[31] Helm ET, Curry SM, De Mille CM, Schweer WP, Burrough ER, Zuber EA, Lonergan SM, Gabler NK (2019). Impact of porcine reproductive and respiratory syndrome virus on muscle metabolism of growing pigs. J Anim Sci.

[32] Nogueira MG, Collins AM, Donahoo M, Emery D (2013). Immunological responses to vaccination following experimental *Lawsonia intracellularis* virulent challenge in pigs. Vet Microbiol.

[33] Husband AJ, Kramer DR, Bao S, Sutherland RM, Beagley KW (1996). Regulation of mucosal IgA responses in vivo: cytokines and adjuvants. Vet Immunol Immunopathol.

[34] Pasetti MF, Simon JK, Sztein MB, Levine MM (2011). Immunology of gut mucosal vaccines. Immunol Rev.

[35] Huan YW, Bengtsson RJ, MacIntyre N, Guthrie J, Finlayson H, Smith SH, Archibald AL, Ait-Ali T (2017). *Lawsonia intracellularis* exploits beta-catenin/Wnt and Notch signalling pathways during infection of intestinal crypt to alter cell homeostasis and promote cell proliferation. PLoS One.

[36] Alexander RT (2020). Claudin-15 is not a drag!. Acta Physiol (Oxf).

[37] Tang M, Zhao Y, Liu NJ, Chen E, Quan Z, Wu XH, Luo CL (2017). Overexpression of HepaCAM inhibits bladder cancer cell proliferation and viability through the AKT/FoxO pathway. J Cancer Res Clin Oncol.

[38] Helm ET, Curry SM, Schwartz KJ, Lonergan SM, Gabler NK (2019). Mycoplasma hyopneumoniae-*Lawsonia intracellularis* dual challenge modulates intestinal integrity and function1. J Anim Sci.

[39] Takeuchi A (1967). Electron microscope studies of experimental *Salmonella* infection. I. Penetration into the intestinal epithelium by *Salmonella* typhimurium. Am J Pathol.

[40] Clark MA, Jepson MA, Simmons NL, Hirst BH (1994). Preferential interaction of *Salmonella* typhimurium with mouse Peyer’s patch M cells. Res Microbiol.

[41] Muller AJ, Kaiser P, Dittmar KE, Weber TC, Haueter S, Endt K, Songhet P, Zellweger C, Kremer M, Fehling HJ, Hardt WD (2012). *Salmonella* gut invasion involves TTSS-2-dependent epithelial traversal, basolateral exit, and uptake by epithelium-sampling lamina propria phagocytes. Cell Host Microbe.

[42] Beloeil PA, Fravalo P, Fablet C, Jolly JP, Eveno E, Hascoet Y, Chauvin C, Salvat G, Madec F (2004). Risk factors for *Salmonella enterica* subsp. *enterica* shedding by market-age pigs in French farrow-to-finish herds. Prev Vet Med.

[43] Leite FLL, Singer RS, Ward T, Gebhart CJ, Isaacson RE (2018). Vaccination against *Lawsonia intracellularis* decreases shedding of *Salmonella enterica* serovar Typhimurium in co-infected pigs and alters the gut microbiome. Sci Rep.

[44] Leite FL, Vasquez E, Gebhart CJ, Isaacson RE (2019). The effects of *Lawsonia intracellularis*, *Salmonella enterica* serovar Typhimurium and co-infection on IL-8 and TNFalpha expression in IPEC-J2 cells. Vet Microbiol.

[45] Mullebner A, Dorighello GG, Kozlov AV, Duvigneau JC (2017). Interaction between mitochondrial reactive oxygen species, heme oxygenase, and nitric oxide synthase stimulates phagocytosis in macrophages. Front Med.

[46] Weiss G, Schaible UE (2015). Macrophage defense mechanisms against intracellular bacteria. Immunol Rev.

[47] Broz P, Monack DM (2011). Molecular mechanisms of inflammasome activation during microbial infections. Immunol Rev.

[48] Man SM, Kanneganti TD (2015). Regulation of inflammasome activation. Immunol Rev.

[49] Johnson RW (1997). Inhibition of growth by pro-inflammatory cytokines: an integrated view. J Anim Sci.

[50] Li Y, Song Z, Kerr KA, Moeser AJ (2017). Chronic social stress in pigs impairs intestinal barrier and nutrient transporter function, and alters neuro-immune mediator and receptor expression. PLoS One.

[51] Erben U, Loddenkemper C, Doerfel K, Spieckermann S, Haller D, Heimesaat MM, Zeitz M, Siegmund B, Kuhl AA (2014). A guide to histomorphological evaluation of intestinal inflammation in mouse models. Int J Clin Exp Pathol.

[52] McOrist S, Gebhart CJ, Zimmerman JJ, Karriker LA, Ramirez A, Schwartz KJ, Stevenson GW (2012). Proliferative enteropathy. Diseases of swine.

[53] Visscher C, Mischok J, Sander S, Schmicke M, Peitzmeier EU, von dem Busche I, Rohn K, Kamphues J (2018). Nutrient digestibility, organ morphometry and performance in vaccinated or non-vaccinated *Lawsonia intracellularis* infected piglets. BMC Vet Res.

[54] Badawy AM, Campbell RM, Cuthbertson DP, Fell BF (1957). Changes in the intestinal mucosa of the sheep following death by humane killer. Nature.

[55] Krawielitzki K, Zebrowska T, Schadereit R, Kowalczyk J, Hennig U, Wünsche J, Herrmann U (1990). Determining of nitrogen absorption and nitrogen secretion in different sections of the pig’s intestine by digesta exchange between 15N labelled and unlabelled animals. Arch Anim Nutr.

[56] Schweer WP, Burrough ER, Patience JF, Kerr BJ, Gabler NK (2019). Impact of *Brachyspira hyodysenteriae* on intestinal amino acid digestibility and endogenous amino acid losses in pigs. J Anim Sci.

[57] Rakhshandeh A, Dekkers JC, Kerr BJ, Weber TE, English J, Gabler NK (2012). Effect of immune system stimulation and divergent selection for residual feed intake on digestive capacity of the small intestine in growing pigs. J Anim Sci.

[58] Schweer W, Schwartz K, Patience JF, Karriker L, Sparks C, Weaver M, Fitzsimmons M, Burkey TE, Gabler NK (2017). Porcine reproductive and respiratory syndrome virus reduces feed efficiency, digestibility, and lean tissue accretion in grow-finish pigs. Transl Anim Sci.

[59] Bates JM, Akerlund J, Mittge E, Guillemin K (2007). Intestinal alkaline phosphatase detoxifies lipopolysaccharide and prevents inflammation in zebrafish in response to the gut microbiota. Cell Host Microbe.

[60] Lazzeri E, Peired A, Ballerini L, Lasagni L (2012). Adult stem cells in tissue homeostasis and disease. Curr Front Persp Cell Biol.

[61] Vanuytsel T, Senger S, Fasano A, Shea-Donohue T (2013). Major signaling pathways in intestinal stem cells. Biochim Biophys Acta.

[62] MacDonald BT, Tamai K, He X (2009). Wnt/beta-catenin signaling: components, mechanisms, and diseases. Dev Cell.

[63] van de Wetering M, Sancho E, Verweij C, de Lau W, Oving I, Hurlstone A, van der Horn K, Batlle E, Coudreuse D, Haramis AP, Tjon-Pon-Fong M, Moerer P, van den Born M, Soete G, Pals S, Eilers M, Medema R, Clevers H (2002). The beta-catenin/TCF-4 complex imposes a crypt progenitor phenotype on colorectal cancer cells. Cell.

[64] Sansom OJ, Reed KR, Hayes AJ, Ireland H, Brinkmann H, Newton IP, Batlle E, Simon-Assmann P, Clevers H, Nathke IS, Clarke AR, Winton DJ (2004). Loss of Apc in vivo immediately perturbs Wnt signaling, differentiation, and migration. Genes Dev.

[65] Andreu P, Peignon G, Slomianny C, Taketo MM, Colnot S, Robine S, Lamarque D, Laurent-Puig P, Perret C, Romagnolo B (2008). A genetic study of the role of the Wnt/beta-catenin signalling in Paneth cell differentiation. Dev Biol.

[66] Reya T, Clevers H (2005). Wnt signalling in stem cells and cancer. Nature.

[67] Riccio O, van Gijn ME, Bezdek AC, Pellegrinet L, van Es JH, Zimber-Strobl U, Strobl LJ, Honjo T, Clevers H, Radtke F (2008). Loss of intestinal crypt progenitor cells owing to inactivation of both Notch1 and Notch2 is accompanied by derepression of CDK inhibitors p27Kip1 and p57Kip2. EMBO Rep.

[68] Kazanjian A, Noah T, Brown D, Burkart J, Shroyer NF (2010). Atonal homolog 1 is required for growth and differentiation effects of notch/gamma-secretase inhibitors on normal and cancerous intestinal epithelial cells. Gastroenterology.

[69] Fre S, Pallavi SK, Huyghe M, Lae M, Janssen KP, Robine S, Artavanis-Tsakonas S, Louvard D (2009). Notch and Wnt signals cooperatively control cell proliferation and tumorigenesis in the intestine. Proc Natl Acad Sci USA.

