# Supplementary material for: Lawsonia intracellularis infected enterocytes lack sucrase-isomaltase which contributes to reduced pig digestive capacity
Source: Vet Res. 2021 Jun 19;52:90. doi: 10.1186/s13567-021-00958-2 (PMC8214296; doi:10.1186/s13567-021-00958-2)
Supplement: Supplementary file 1 — Additional file 1 Diet composition, as fed. [file 13567_2021_958_MOESM1_ESM.docx]

**Additional file 1.** Diet composition, as fed

| Ingredient | % in diet |
| --- | --- |
| Corn | 59.71 |
| Soybean meal | 26.73 |
| Corn DDGS^1^ | 10.00 |
| Soybean oil | 0.50 |
| Salt | 0.35 |
| Monocalcium phosphate, 21% | 0.68 |
| Limestone | 1.14 |
| L-Lysine HCl | 0.20 |
| Vitamin premix^2^ | 0.15 |
| Trace mineral premix^3^ | 0.15 |
| Titanium dioxide | 0.40 |
| *Calculated composition* |  |
| ME, kcal/kg | 3,328 |
| SID Lysine, %^4^ | 1.02 |
| *Analyzed composition* |  |
| DM, % | 86.39 |
| CP, % | 18.65 |
| GE, kcal/kg | 3,817 |

^1^DDGS = distiller’s dried grains with solubles.

^2^Provided per kilogram of diet: 6,125 IU vitamin A, 700 IU vitamin D_3_, 50 IU vitamin E, 30 mg vitamin K, 0.05 mg vitamin B_12_, 11 mg riboflavin, 56 mg niacin, and 27 mg pantothenic acid.

^3^Provided per kilogram of diet: 22 mg Cu (as CuSO_4_), 220 mg Fe (as FeSO_4_), 0.4 mg I (as Ca(IO_3_)_2_), 52 mg Mn (as MnSO_4_), 220 mg Zn (as ZnSO_4_), and 0.4 mg Se (as Na_2_SeO_3_).

^4^SID = standardized ileal digestibility.
