# Supplementary material for: Lawsonia intracellularis infected enterocytes lack sucrase-isomaltase which contributes to reduced pig digestive capacity
Source: Vet Res. 2021 Jun 19;52:90. doi: 10.1186/s13567-021-00958-2 (PMC8214296; doi:10.1186/s13567-021-00958-2)
Supplement: Supplementary file 2 — Additional file 2 Primer sequences. [file 13567_2021_958_MOESM2_ESM.docx]

**Additional file 2.** Primer sequences.

| **Gene** | **Sense (5'-3') - forward** | | **Antisense (5'-3') - reverse** |
| --- | --- | --- | --- |
| ACTB | | CCAACATTGGTTATGGGAGCAA | GGAAGAGACGTTGTGAGCAA |
| ATOH | | GAACGGGGTACAGAAGCAAA | TGGACAGCTTCTTGTCGTTG |
| HES1 | | AAGGCGGACATTCTGGAAAT | CCTCGTTCATGCACTCACTG |
| IAP | | AACCGCAGGACATTCCTTCA | TTCATGTCTGCCGGCTCAA |
| MUC2 | | CTGTGTGGGGCCTGACAA | AGTGCTTGCAGTCGAACTCA |
| P27^kip1^ | | TTGGCTCGATACTGAGGGGA | ACCTGCCACAGACGGTAATG |
| SI | | GAACTCACAAAAAGAACTGAAGGATTAC | GCAAAACAGATGATCCATCACTAAGA |
| WNT3 | | GAGTGCCAACACCAGTTCC | AGTCACAGCGAAGGCAACTC |
| β-catenin | | TCTCCCAGCAACATACGCAG | TCTCCCAGCAACATACGCAG |
