# Supplementary material for: Lawsonia intracellularis infected enterocytes lack sucrase-isomaltase which contributes to reduced pig digestive capacity
Source: Vet Res. 2021 Jun 19;52:90. doi: 10.1186/s13567-021-00958-2 (PMC8214296; doi:10.1186/s13567-021-00958-2)
Supplement: Supplementary file 3 — Additional file 3 Macroscopic lesion severity. [file 13567_2021_958_MOESM3_ESM.docx]

**Additional file 3.** Macroscopic lesion severity in non-infected pigs (NC), *Lawsonia intracellularis* inoculated pigs (PC), and vaccinated *Lawsonia intracellularis* inoculated pigs (VAC)

|  | NC | PC | VAC | SEM | *P*-value | NC vs. PC | NC vs. VAC | PC vs. VAC |
| --- | --- | --- | --- | --- | --- | --- | --- | --- |
| Jejunum | 0.00 | 0.33 | 0.00 | 0.333 | 1.000 | 0.576 | 1.000 | 0.576 |
| Ileum | 0.00 | 1.92 | 0.25 | 0.468 | <0.001 | 0.003 | 0.318 | 0.018 |
| Cecum | 0.08 | 1.00 | 0.25 | 0.275 | 0.004 | 0.010 | 0.531 | 0.068 |
| Colon | 0.33 | 0.83 | 0.75 | 0.298 | 0.422 | 0.466 | 0.465 | 0.995 |
|  |  |  |  |  |  |  |  |  |
| Ileal Lesion length, cm | 0.00 | 118.4 | 2.97 | 37.23 | 0.001 | 0.007 | 0.318 | 0.045 |

Macroscopic lesions were evaluated on a 0-4 scale as follows: 0 if no gross lesion; 1 if mild edema and hyperemia of mucosa or serosa; 2 if edema, hyperemia, reticulated serosa and mucosa (thickening); 3 if edema, hyperemia, reticulated serosa and mucosa and gross thickening of the mucosa; and 4 if severe thickening mucosal hemorrhaging or necrosis. Presented as means ± SEM, n=12 pigs/treatment.
