# Supplementary material for: Lawsonia intracellularis infected enterocytes lack sucrase-isomaltase which contributes to reduced pig digestive capacity
Source: Vet Res. 2021 Jun 19;52:90. doi: 10.1186/s13567-021-00958-2 (PMC8214296; doi:10.1186/s13567-021-00958-2)
Supplement: Supplementary file 4 — Additional file 4 Microscopic lesion severity. [file 13567_2021_958_MOESM4_ESM.docx]

**Additional file 4.** Microscopic lesion severity in non-infected pigs (NC), *Lawsonia intracellularis* inoculated pigs (PC), and vaccinated *Lawsonia intracellularis* inoculated pigs (VAC)

|  |  | NC | PC | VAC | SEM | *P*-value | NC vs. PC | NC vs. VAC | PC vs. VAC |
| --- | --- | --- | --- | --- | --- | --- | --- | --- | --- |
| Ileum | |  |  |  |  |  |  |  |  |
|  | Inflammation^1^ | 0.25 | 2.08 | 0.83 | 0.288 | <0.001 | 0.001 | 0.081 | 0.011 |
|  | Crypt^2^ | 0.00 | 2.42 | 0.42 | 0.229 | <0.001 | <0.001 | 0.037 | <0.001 |
|  | Distribution^3^ | 0.25 | 2.33 | 1.08 | 0.189 | <0.001 | <0.001 | 0.044 | 0.006 |
|  | IHC^4^ | 0.00 | 2.16 | 0.50 | 0.386 | <0.001 | <0.001 | 0.002 | 0.003 |
|  | Overall^5^ | 0.50 | 9.00 | 2.83 | 0.992 | <0.001 | <0.001 | 0.028 | 0.001 |
| Cecum | |  |  |  |  |  |  |  |  |
|  | Inflammation^1^ | 1.41 | 1.50 | 1.08 | 0.151 | 0.149 | 0.915 | 0.289 | 0.158 |
|  | Crypt^2^ | 0.17 | 0.33 | 0.25 | 0.188 | 0.829 | 0.835 | 0.875 | 0.993 |
|  | Distribution^3^ | 2.00 | 1.92 | 1.92 | 0.149 | 0.914 | 0.931 | 0.997 | 0.929 |
|  | IHC^4^ | 0.00 | 0.42 | 0.08 | 0.192 | 0.051 | 0.082 | 0.577 | 0.286 |
|  | Overall^5^ | 3.59 | 4.17 | 3.33 | 0.423 | 0.398 | 0.610 | 0.959 | 0.372 |
| Colon | |  |  |  |  |  |  |  |  |
|  | Inflammation^1^ | 1.00 | 1.92 | 1.17 | 0.193 | 0.004 | 0.009 | 0.768 | 0.028 |
|  | Crypt^2^ | 0.08 | 1.08 | 0.42 | 0.260 | 0.003 | 0.003 | 0.286 | 0.111 |
|  | Distribution^3^ | 1.67 | 2.17 | 1.67 | 0.167 | 0.192 | 0.315 | 0.978 | 0.217 |
|  | IHC^4^ | 0.00 | 1.00 | 0.17 | 0.276 | 0.001 | 0.003 | 0.317 | 0.029 |
|  | Overall^5^ | 2.50 | 5.50 | 3.00 | 0.669 | 0.002 | 0.004 | 0.807 | 0.013 |
| Total Score^6^ | | 6.58 | 18.68 | 9.17 | 1.170 | <0.001 | <0.001 | 0.178 | <0.001 |

Presented as means ± SEM

^1^Inflammation: 0) none/minimal, 1) mild, 2) moderate, and 3) severe

^2^Proliferation of crypts: 0) none/minimal, 1) mild, 2) moderate, and 3) severe

^3^Lesion distribution: 0) none, 1) focal, 2) multifocal, and 3) diffuse

^4^IHC: 0) no antigen, 1) 1-25% of crypts, 2) 26-50% of crypts, 3) 51-75% of crypts, 4) >75% of crypts

^5^Overall = cumulative scores for that segment. Max score possible = 13

^6^Total score = additive scores for all segments. Max score possible = 36
