# Supplementary material for: Lawsonia intracellularis infected enterocytes lack sucrase-isomaltase which contributes to reduced pig digestive capacity
Source: Vet Res. 2021 Jun 19;52:90. doi: 10.1186/s13567-021-00958-2 (PMC8214296; doi:10.1186/s13567-021-00958-2)
Supplement: Supplementary file 5 — Additional file 5 Ex vivo intestinal integrity and function parameters. [file 13567_2021_958_MOESM5_ESM.docx]

**Additional file 5.** *Ex vivo* intestinal integrity and function parameters of non-infected pigs (NC), *Lawsonia intracellularis* inoculated pigs (PC), and vaccinated *Lawsonia intracellularis* inoculated pigs (VAC).

|  |  | Treatment | | |  |  |
| --- | --- | --- | --- | --- | --- | --- |
|  |  | NC | PC | VAC | SEM | *P*-Value |
| Ileum | |  |  |  |  |  |
|  | Transepithelial resistance, Ω x cm^2^ | 55.59 | 57.75 | 59.16 | 5.420 | 0.877 |
|  | Glucose transport^1^ | 0.94^b^ | 1.66^ab^ | 3.72^a^ | 0.667 | 0.014 |
|  | Glutamine Transport^1^ | 0.22 | 0.41 | 0.24 | 0.131 | 0.508 |
|  | FD4 permeability^2^ | 63.55 | 40.07 | 52.34 | 0.164 | 0.210 |
|  | *S*. Typhimurium translocation^3^ | 4.40 | 4.44 | 3.91 | 0.289 | 0.362 |
| Colon | |  |  |  |  |  |
|  | Transepithelial resistance, Ω x cm^2^ | 26.34 | 32.08 | 31.2 | 2.266 | 0.189 |
|  | FD4 permeability^2^ | 282.4 | 209.4 | 196.2 | 38.98 | 0.234 |
|  | *S*. Typhimurium translocation^3^ | 4.48 | 4.44 | 3.87 | 0.297 | 0.266 |

^a,b^Means with differing superscripts differ significantly at *P* < 0.05. Data represents 12 pigs/treatment.

^1^μA, active absorption calculated by subtracting μA before substrate (glucose or glutamine) from μA after substrate addition.

^2^Mucosal to serosal translocation of Fluorescein isothiocyanate–dextran 4kDa (FD4)

^3^*S*. Typhimurium mucosal to serosal translocation, log_10_CFU/mL
