# Supplementary material for: Lawsonia intracellularis infected enterocytes lack sucrase-isomaltase which contributes to reduced pig digestive capacity
Source: Vet Res. 2021 Jun 19;52:90. doi: 10.1186/s13567-021-00958-2 (PMC8214296; doi:10.1186/s13567-021-00958-2)
Supplement: Supplementary file 6 — Additional file 6 Ileal and colonic mitochondrial parameters. [file 13567_2021_958_MOESM6_ESM.docx]

**Additional file 6.** Ileal and colonic mitochondrial parameters in non-infected pigs (NC), *Lawsonia intracellularis* inoculated pigs (PC), and vaccinated *Lawsonia intracellularis* inoculated pigs (VAC)

|  |  | Treatment | | |  |  |
| --- | --- | --- | --- | --- | --- | --- |
|  |  | NC | PC | VAC | SEM | *P*-Value |
| Ileal mitochondrial respiration | |  |  |  |  |  |
|  | Basal^1^ | 147.8 | 198 | 163 | 24.87 | 0.344 |
|  | ADP, State 3^1^ | 1110 | 1211 | 1217 | 120 | 0.778 |
|  | Oligomycin, State 4_o_^1^ | 23.67 | 33.69 | 68.63 | 26.6 | 0.398 |
|  | FCCP, State 3_μ_^1^ | 1150 | 866 | 934 | 111.7 | 0.188 |
|  | RCR, State 3_μ_/4_o_ | 8.16 | 5.18 | 6.17 | 0.911 | 0.077 |

^1^pmol O_2_/min
